# Assessment of industrial fault diagnosis using rough approximations of fuzzy hypersoft sets

**DOI:** 10.1371/journal.pone.0329185

**Published:** 2025-09-09

**Authors:** Muhammad Abdullah, Khuram Ali Khan, Atiqe Ur Rahman, Rostin Matendo Mabela

**Affiliations:** 1 Department of Mathematics, University of Sargodha, Sargodha, Pakistan; 2 Department of Mathematics, University of Management and Technology, Lahore, Pakistan; 3 Department of Maths and Computer Science, Faculty of Science, University of Kinshasa, Kinshasa, The Democratic Republic of the Congo; University of Hamburg: Universitat Hamburg, GERMANY

## Abstract

Reliable and timely fault diagnosis is critical for the safe and efficient operation of industrial systems. However, conventional diagnostic methods often struggle to handle uncertainties, vague data, and interdependent multi-criteria parameters, which can lead to incomplete or inaccurate results. Existing techniques are limited in their ability to manage hierarchical decision structures and overlapping information under real-world conditions. To address these limitations, this paper proposes a novel diagnostic framework based on Hypersoft Fuzzy Rough Set (HSFRS) theory.This hybrid approach integrates the flexibility of hypersoft sets for modeling multi-parameter relationships, the strength of fuzzy logic in handling vagueness, and the approximation capabilities of rough set theory to manage data uncertainty. Using a pseudo fuzzy binary relation, we define lower and upper approximation operators for fuzzy subsets within the parameter space. An enhanced Bingzhen and Weimin model-based decision-making algorithm is developed to support intelligent diagnosis. A case study involving a conveyor belt system is presented, evaluating eight fault states using five primary parameters and twenty sub-parameters. The results confirm the robustness, interpretability, and effectiveness of the proposed model in complex industrial scenarios by ranking the states based on fuzzy hypersoft closeness degrees.

## 1 Introduction

In machine health management, fault diagnosis plays a crucial role in establishing the relationship between the machine’s health status and monitoring data, including sounds, vibrations, and other signals. This partnership is crucial because it guarantees prompt maintenance, enables the early identification of possible problems, and guards against equipment failures. This process has historically placed a great deal of reliance on the vast experience and specialized knowledge of engineers. When an engine malfunctions, skilled engineers can often identify the problem by listening to unusual noises or by analyzing vibration signals and identifying problems such as bearing failures with sophisticated signal processing techniques. Because of their experience, they can accurately assess a machine’s condition by interpreting subtle signs and data [1, 2]. Automated techniques to improve diagnostic accuracy and optimize maintenance procedures are becoming more and more popular among machine users in engineering contexts. The need to cut downtime and boost the effectiveness of troubleshooting machines is what is driving this change. There is a growing expectation that fault diagnosis will become more intelligent and self-sufficient with advances in artificial intelligence (AI). AI technologies can accurately identify the health status of machines by automatically detecting and interpreting signals, such as unusual noises or vibrations. By reducing human error and expediting the diagnosis process, this automation offers a more dependable and effective method of preserving machine health [3–5]. It is possible to think fault diagnosis as a multi-criteria decision-making (MCDM) problem [6]. When making decisions that require establishing or addressing planning and determination issues under multiple criteria, MCDM is an essential component of the process [7]. It does this by quantifying the significance of different criteria for particular goals, which helps managers make well-informed decisions. Since these variables can affect a project’s success on multiple levels–economic, social, cultural, and environmental–MCDM methods excel at taking into account these multifaceted aspects of problems. A specialized method of decision-making called MCDM entails the selection of options, the establishment of criteria levels, the ranking of alternatives, and the depiction of the various behaviors of the options [8, 9]. Our lives are made easier by industrial systems in every way. Energy waste and financial loss are possible outcomes of faults. Finding the location of defects and detecting their beginning are crucial engineering jobs [10]. Failure and fault are not the same thing since while a system may function, a system failure will prevent it from working. The unapproved departure of a single system or a component parameter from the standard state is the fault [11]. The fault could result in energy waste, a reduction in system longevity or efficiency, or perhaps the system failing completely. Moreover, the flaw can cause the system’s physical components to be destroyed. Supervisory functions aid with maintenance and damage limitation avoidance by indicating undesirable process states. Monitoring, automated protection, and supervision with fault diagnostics are examples of supervisory functions [12]. The task of diagnosing faults is difficult because of the inherent uncertainties that result from multiple factors [13, 14]. These uncertainties include imperfect knowledge of the system’s condition, noise and anomalies in sensor data, and the inherent complexity of contemporary systems, which frequently consist of a large number of interconnected parts. Furthermore, there are many different circumstances in which systems can operate, which makes it challenging to differentiate between typical behavioral fluctuations and real faults. Systems are represented by simplified models that might not fully capture all the subtleties, which increases uncertainty. To make matters more complicated, it is possible that some of the parameters in these models are not well-known. Errors, prejudices, and subjective interpretations are examples of human factors that add to the uncertainty. For fault diagnosis to be effective, therefore, sophisticated techniques that can handle noisy, imprecise, and incomplete data as well as take into consideration the complexities and variations in system behavior are needed [15, 16].

The ideas like fuzzy set (FS) [17], rough set (RS) [18], fuzzy rough set (FRS) [19] and generalized fuzzy rough set [20] can efficiently handle the issues related to inherent uncertainties, impreciseness, and incompleteness. To equip these structures with parameterization context, Molodtsov [21] initiated the idea of soft set (SS) which employs an approximate mapping to assist the decision-makers in evaluating the alternatives concerning particular parameters. Considering the idea of soft elements and soft members, Saeed *et al*. [22] introduced algebraic structures for the SS environment. Sezgin *et al*. [23] discussed the different properties and theorems of a new SS operation known as complementary soft binary piecewise intersection. Cagman *et al*. [24] defined the fuzzy soft set (FSS) theory and discussed its properties. Additionally, they defined the aggregation operator of FSS and applied it in human resource management to assess the method’s validity. The concept of SS is ill-suited for scenarios requiring a multi-argument domain, such as the allocation of parameters and their sub-parametric values collectively. To solve this issue, Smarandache [25] introduced the hypersoft set (HSS), which is a modified approximated mapping that considers multiple arguments as opposed to just one argument or parameter. Through the consideration of parameter and sub-parameter indeterminacy, he [26] further introduced some new types of SS and HSS. Saeed *et al*. [27] defined several operations, proved theorems, and covered several other HSS properties to improve the adaptability and applicability of HSS. Rahman *et al*. [28] discussed the supplier’s evaluation by considering operational risks using hypersoft mappings. Debnath [29] explored weightage operators of fuzzy hypersoft set (FHSS) for decision-making scenarios. Saeed *et al*. [30] formulated the entropy and similarity measures for FHSS and discussed renewable energy resource evaluation. Saeed *et al*. [31] looked into the various properties and results of FHSS graphs. Kamacı and Saqlain [32] developed n-ary fuzzy hypersoft expert set by integrating FHSS and expert set to combine multi-decisive opinions and multi-argument-based approximate mapping. Al-Quran *et al*. [33] discussed the various aspects and operations of bipolarity in the FHSS environment and car evaluation problems based on the aggregation operators of bipolar FHSS. Ullah and Shah [34] developed matrix theory for FHSS and discussed decision-making problems based on this theory. Rahman *et al*. [35] investigated the susceptibility of liver diseases based on the uncertain nature of multi-argument tuples in the FHSS environment. Ahsan *et al*. [36] explored the procedure for optimized novel technology utilization by incorporating entropy measures, similarity measures, and TOPSIS of FHSS. Asaad *et al*. [37] discussed the several features and operations of bipolarity in the FHSS environment to evaluate soft engineers for a company.

Rough soft set (RSS) [38–40] is a type of hybrid mathematical model designed to manage imprecision, ambiguity, and uncertainty in the interpretation of data. It combines the ideas of RS and SS. When limits are not clearly defined, RS concentrates on approximating sets, but SS offers a flexible framework for addressing uncertainty by including parameterization. An element may only partially belong to an RSS if the lower and higher approximations are established using rough and SS principles. In information systems and decision-making processes where data ambiguity and partial truths are frequent, this paradigm is quite helpful. Sarwar *et al*. [41] employed the rough soft approximations of graph and hypergraph for selecting authors for different areas of natural and social sciences. El-Bably *et al*. [42] discussed the clinical assessment of Chikungunya using the aggregations of RSS. Sun and Ma [43] introduced the idea of the soft fuzzy rough set (SFRS) and discussed its lower and upper approximations based on pseudo fuzzy binary relations. Meng *et al*. [44], Hu *et al*. [45], Feng *et al*. [46] and Liu *et al*. [47] made rich contributions in the field of SFRS. Zhang *et al*. [48] discussed the parameter reduction in the context of SFRS. Bingzhen and Weimin [49] evaluated an optimized emergency plan for untraditional emergent situations based on SFRS. Rahman *et al*. [50] discussed supplier evaluation problems for the construction industry by incorporating triangular fuzzy numbers and FHSS. Azim *et al*. [51] explored sophisticated methods of collecting information and how they may be used to comprehend how customers and employees behave in particular places. In order to choose the best technological solution, they used the *q*-spherical fuzzy rough TOPSIS approach, incorporating three crucial parameters: parameter *q* (where q≥1), upper set approximation, and lower set approximation. Soni and Mehta [52] developed fuzzy logic controllers (FLC) and fuzzy clustering means (FCM) for the diagnosis and prognosis of problems caused by different loads. They imitated Fuzzy clustering is a method that analyses the insulation’s health by forming different clusters.

### 1.1 Research gap, challenges and questions

Despite the contributions of the aforementioned studies, many existing approaches suffer from significant limitations. Traditional rough set models often assume precise parameter values and cannot effectively address fuzzy uncertainty. Similarly, fuzzy and soft computing-based models frequently lack the structural flexibility needed to handle hierarchical or interrelated attributes. Moreover, few studies integrate multiple uncertainty-handling tools to simultaneously capture data granularity, ambiguity, and overlapping decision parameters. These gaps highlight the need for an advanced hybrid framework, such as the one proposed in this paper, that can more comprehensively support fault diagnosis in industrial systems. A complex mathematical framework designed to manage ambiguity, imprecision, and uncertainty in data is represented by rough approximations of fuzzy hypersoft sets. This thorough investigation draws attention to these deficiencies and offers a research plan.

1. Rough approximations of FHS are still not well understood or handled, despite major advances in FSS and ROS.
2. The extant research often concentrates on RS or HSS alone, but the combination of both ideas is yet largely unexplored.
3. The formal integration of FSS, HSS, and FSS is severely lacking. Although there are sporadic research integrating various notions, there isn’t a thorough and uniform framework available.
4. Rough approximation properties and their mathematical formulation in the framework of FHSS are not completely explored. This calls for exacting definitions, axiomatic bases, and behavior-governing theorems.

Because of their qualities, the techniques of RS and FHSS as well as their expansions, are very dependable and adaptable. This was covered in the study above. Nevertheless, the proposed study on the Hypersoft Fuzzy Rough Set (HSFRS) model for industrial fault diagnosis is guided by the following research questions:

1. Can the HRS model improve the accuracy of fault diagnosis in industrial systems by effectively handling multi-layered uncertainties and complex interdependencies among fault criteria?
2. How does the HRS model’s capability to capture multi-attribute dependencies impact the robustness of fault classification under fluctuating operational conditions?
3. What is the comparative performance of the HRS model against traditional rough set and SS models in identifying faults accurately in high-dimensional industrial datasets?

These questions are intended to investigate whether the HRS model can address specific limitations of current methods by enhancing fault detection accuracy, adaptability, and classification precision in complex industrial environments. The study hypothesizes that the HRS model will outperform traditional models in handling uncertainty and dependency complexity, resulting in more accurate and robust industrial fault diagnosis. Experts have lost a great deal of information throughout the decision-making process because of these four questions, which are the main components of any decision-making process. Furthermore, based on our assessments and information, we discovered that the HFRS approach has not yet been produced due to a number of issues and obstacles. In order to define the HFRS technique, we had to merge the theories of fuzzy rough sets and HSS.

### 1.2 Novelty of the proposed study

In every aspect of life, industrial systems are useful to us. Errors have the potential to cause financial loss and energy waste. Important work in engineering includes locating flaws and detecting their beginning. A significant category of fault diagnosis and detection techniques makes use of the monitored system’s mathematical model. But for the majority of practical industrial engineering applications, the characteristics needed for mathematical modeling are either unavailable or restricted. One of the key methods for fault diagnosis and identification is observer-based fault diagnosis. This work presents a novel mathematical model, HRS, which advances traditional rough set theory by integrating HSS principles to better address complex, multi-layered uncertainty in decision-making. The originality lies in extending rough set theory to include hypersoft sets, allowing for the handling of multi-attribute dependencies and overlapping data categories that are difficult to address with conventional rough or soft sets. Unlike existing models, this HRS approach introduces a flexible granularity to capture intricate interrelations among criteria and sub-criteria, supporting nuanced decision-making under conditions where criteria are interdependent or change over time. Additionally, the model incorporates a refined approximation strategy that improves classification accuracy by using HSS-based granular partitions, making it a robust tool for applications such as fault diagnosis in dynamic environments. This framework’s ability to manage layered uncertainties and attribute granularity positions it as a significant advancement in rough set-based decision models.

### 1.3 Salient contributions

Although various fault diagnosis techniques based on fuzzy logic, rough sets, and soft computing have been proposed, these methods often fall short in managing hierarchical parameter structures, overlapping attributes, and multiple sources of uncertainty. Most existing models either lack the ability to handle fuzzy granularity in multi-criteria data or cannot represent complex relationships among parameters effectively. To bridge this gap, we propose a novel diagnostic framework that integrates Hypersoft Sets with Fuzzy Rough Set theory, allowing for a more comprehensive and flexible decision-making structure. Our approach introduces hypersoft fuzzy rough approximation operators using pseudo fuzzy binary relations and incorporates them into an enhanced decision-making algorithm. A case study on conveyor belt fault states validates the proposed model’s effectiveness, robustness, and superior ranking ability under uncertainty. The summary of some noteworthy contributions of this study is given as

1. The suggested structures are seen to be the most effective means of examining the intricacies of fault diagnosis. Their ability to manage a wider range of membership grades is a advantage, particularly when working with attributes that have several sub-values. Without a doubt, these frameworks offer the greatest means of investigating the topic of fault diagnosis. Their ability to cater to a broad range of membership classes is what makes them successful, especially when handling attributes with several sub-values.
2. Expert assessments are a helpful instrument for showcasing practical applications and showing the significance of the recommended course of action. The assessments and observations of subject-matter experts are used to show how the methodology can be successfully used in real-world scenarios.
3. Moreover, a sensitivity analysis is conducted to examine the impact of altering the weights assigned to the significant variables throughout the sorting process. This research allows for a better understanding of how changes to these weights impact the ultimate outcome or option ranking. By gradually adjusting the weights and monitoring the ensuing changes in ranks, researchers can gain greater insight into the durability and dependability of the separating approach used, which enhances the precision and reliability of the decision making process.
4. Despite being primarily focused on industrial fault diagnosis, the framework’s adaptability suggests that it may also be utilized in other fields where multi-criteria decision-making under uncertainty is necessary, like environmental monitoring, financial analysis, and medical diagnosis.

The structure of this paper is as follows: The fundamental ideas required for this paper, such that fuzzy soft sets, fuzzy rough sets, Pawlak rough sets, soft sets, and HSS are briefly introduced in Sect 2. The hypersoft fuzzy rough set model is established in Sect 3 along with a detailed discussion of its characteristics. The concept and evaluation process for fault diagnosis in industrial systems based on hypersoft fuzzy rough sets are presented in Sect 4. Simultaneously, we suggest an algorithm for this fault diagnosis evaluation approach. In Sect 5, we examine a numerical example that is applied and confirm the accuracy of the theories and methods put forth in this work. In Sect 6, we draw conclusions from our study and suggest areas for future investigation.

## 2 Fundamental knowledge

In this part, a few fundamental ideas will be covered. Rough approximations are given for the fuzzy set, as well as its soft and hypersoft structures. In this paper universal set will be presented by Ł, collection of fuzzy subsets by ${\hat{𝔽}}^{\overset{˘}{𝔻}}$, where $\overset{˘}{𝔻}={\tilde{\overset{˘}{𝔻}}}_{1}\times {\tilde{\overset{˘}{𝔻}}}_{2}\times {\tilde{\overset{˘}{𝔻}}}_{3}\times ...\times {\tilde{\overset{˘}{𝔻}}}_{n}$, and all the subsets of $\overset{˘}{𝔻}$ by ${𝔓}^{\overset{˘}{𝔻}}.$

**Definition 1.** *[18] Let $\text{Ł},\ddot{Y}$ and $\hat{ℜ}$ be a universal set, set of attributes and an equivalence relation (indiscernibility relation) respectively, each attribute $\hat{\alpha }\in \ddot{Y}$ is a function $\hat{\alpha }:\text{Ł}⟶V_{\hat{\alpha }}$ where $V_{\hat{\alpha }}$ denotes the collection of values known as the attribute domain. Then $ℑ=\left(\text{Ł},\ddot{Y}\right)$ is known as knowledge representation system or information system. Specifically $\hat{\mathbb{Q}}=\left(\text{Ł},\hat{ℜ}\right)$ is called Pawlak approximation space. The equivalence relationship $\hat{ℜ}$ is associated with an information system and is commonly referred to as an indiscernibility relation. In particular if $ℑ=\left(\text{Ł},\ddot{Y}\right)$ is an information system and $\hat{𝕎}\subseteq \ddot{Y}$, then an indiscernibility relation $\hat{ℜ}=ℑ\left(\hat{𝕎}\right)$ can be defined by*

$$\left(\eta ,\mu \right)\in ℑ\left(\hat{𝕎}\right)⟺\hat{\alpha }\left(\eta \right)=\hat{\alpha }\left(\mu \right),\;\forall \;\hat{\alpha }\in \hat{𝕎},$$ (1)

*where η,μ∈Ł and $\hat{\alpha }\left(\eta \right)$ denotes the value of attribute $\hat{\alpha }$ for object *η*.*

The following two operations can be defined using the indiscernibility relation $\hat{ℜ}$.

$${\overset{\leftarrow }{\hat{ℜ}}}_{\mathbb{Z}}=\left\{\eta \in \text{Ł}:{\left[\eta \right]}_{\hat{ℜ}}\subseteq \mathbb{Z}\right\},$$ (2)

$${\vec{\hat{ℜ}}}_{\mathbb{Z}}=\left\{\eta \in \text{Ł}:{\left[\eta \right]}_{\hat{ℜ}}\cap \mathbb{Z}\neq \phi \right\},$$ (3)

*assigning to each subset ℤ⊆Ł the sets ${\overset{\leftarrow }{\hat{ℜ}}}_{\mathbb{Z}}$ and ${\vec{\hat{ℜ}}}_{\mathbb{Z}}$ are said to be $\hat{ℜ}-$ lower and $\hat{ℜ}-$ upper approximation of ℤ respectively. assigning to each subset ℤ⊆Ł the sets ${\overset{\leftarrow }{\hat{ℜ}}}_{\mathbb{Z}}$ and ${\vec{\hat{ℜ}}}_{\mathbb{Z}}$ are said to be $\hat{ℜ}-$ lower and $\hat{ℜ}-$ upper approximation of ℤ respectively.*

**Definition 2.** *[20] Let $\hat{ℜ}$ be a fuzzy relation from Ł to ℚ, where Ł,ℚ≠ϕ are the universal sets. Then, $\hat{𝕌}=\left(\text{Ł},\mathbb{Q},\hat{ℜ}\right)$ is known as a generalized fuzzy approximation space. The lower and upper approximations ${\overset{\leftarrow }{\hat{ℜ}}}_{\hat{𝔹}}$ and ${\vec{\hat{ℜ}}}_{\hat{𝔹}}$ respectively of any set $\hat{𝔹}\in {\hat{𝔽}}^{\text{Ł}}$, w.r.t approximation space $\hat{𝕌}$ are fuzzy sets of Ł. The membership functions for each η∈Ł, defined as*

$${\vec{\hat{ℜ}}}_{\hat{𝔹}}={⋁}_{\mu \in \mathbb{Q}}\left[\hat{ℜ}\left(\eta ,\mu \right)\wedge \hat{𝔹}\left(\mu \right)\right],\;\eta \in \text{Ł},$$ (4)

$${\overset{\leftarrow }{\hat{ℜ}}}_{\hat{𝔹}}={⋀}_{\mu \in \mathbb{Q}}\left[\left(1-\hat{ℜ}\left(\eta ,\mu \right)\right)\vee \hat{𝔹}\left(\mu \right)\right],\;\eta \in \text{Ł}.$$ (5)

*We call the pair $\left({\overset{\leftarrow }{\hat{ℜ}}}_{\hat{𝔹}},{\vec{\hat{ℜ}}}_{\hat{𝔹}}\right)$ a generalized fuzzy rough set.*

**Definition 3.** *[21] Let $2^{\text{Ł}},\text{Ł}$ and $\ddot{Y}$ be collection of all subsets of Ł, set of universe, and attributes respectively. Then, $\left(\hat{\Xi },\ddot{Y}\right)$ is referred to as a SS over Ł, where $\hat{\Xi }:\ddot{Y}⟶2^{\text{Ł}}.$*

Stated differently, a parameterized family of subsets of the universe Ł is referred to as a SS over Ł. For $\varepsilon \in \ddot{Y}$ might be viewed as the collection of ε− approximate elements of the SS $\left(\hat{\Xi },\ddot{Y}\right).$

**Definition 4.** *[24] Let $2^{\text{Ł}},\text{Ł}$ and $\ddot{Y}$ be a collection of all subsets of Ł, set of universe, and attributes respectively. Then, $\left(\tilde{\Lambda },\ddot{Y}\right)$ is known as FSS over Ł, where $\tilde{\Lambda }:\ddot{Y}⟶{\hat{𝔽}}^{\text{Ł}}.$ In general*

$$\tilde{\Lambda }\left(\varepsilon \right)=\left\{\left(\eta ,\tilde{\Lambda }\left(\varepsilon \right)\left(\eta \right)\right)|\eta \in \text{Ł}\right\}.$$ (6)

**Definition 5.** *[25] Let $2^{\text{Ł}}$ and Ł be a collection of all subsets of Ł and set of universe respectively. Given that $\left({\hat{\iota }}^{1},{\hat{\iota }}^{2},{\hat{\iota }}^{3},...,{\hat{\iota }}^{\hat{n}}\right)$, $\hat{n}\geq 1$, be $\hat{n}$ distinct attributes, the order of the relevant attributive elements is as follows $\left({\hat{\overset{˘}{𝔻}}}^{1},{\hat{\overset{˘}{𝔻}}}^{2},{\hat{\overset{˘}{𝔻}}}^{3},...,{\hat{\overset{˘}{𝔻}}}^{\hat{n}}\right)$, with ${\hat{\overset{˘}{𝔻}}}^{\hat{\iota }}\;\cap \;{\hat{\overset{˘}{𝔻}}}^{\hat{j}}=\phi ,\;\forall \;\hat{\iota }\neq \hat{j}$, and $\hat{\iota },\hat{j}\in ℵ.$ Under these circumstances, the pair $\left(\hat{\Xi },\hat{\overset{˘}{𝔻}}\right)$ is known as a HSS and can be represented*

$$\hat{\Xi }:\left(\hat{\overset{˘}{𝔻}}={\hat{\overset{˘}{𝔻}}}^{1}\times {\hat{\overset{˘}{𝔻}}}^{2}\times {\hat{\overset{˘}{𝔻}}}^{3}\times ...\times {\hat{\overset{˘}{𝔻}}}^{\hat{n}}\right)⟶2^{\text{Ł}}.$$ (7)

## 3 Pseudo fuzzy hypersoft rough approximations

The concept of pseudo fuzzy hypersoft rough approximations is introduced in this section.

**Definition 6.** *Let Ł represents the universal set and let $\ddot{Y}=\{\alpha_{1},\alpha_{2},\alpha_{3},...,\alpha_{n}\}$ be a set of attribu-tes. The corresponding attribute values for every attribute $\alpha_{\hat{ı}},\hat{ı}=1,2,3,...,n$ are respectively the sets ${\tilde{\overset{˘}{𝔻}}}_{1},{\tilde{\overset{˘}{𝔻}}}_{2},{\tilde{\overset{˘}{𝔻}}}_{3},...,{\tilde{\overset{˘}{𝔻}}}_{n}$, with ${\tilde{\overset{˘}{𝔻}}}_{\hat{ı}}\cap {\tilde{\overset{˘}{𝔻}}}_{\hat{j}}=\phi ,\;for\;\hat{ı}\neq \hat{j},\;and\;\;\hat{ı},\hat{j}\in ℵ$. Then the pair $\left(\Lambda^{-1},\overset{˘}{𝔻}\right)$ is called a pseudo HSS over Ł iff $\Lambda^{-1}:⟶{𝔓}^{\overset{˘}{𝔻}}$, where $\overset{˘}{𝔻}={\tilde{\overset{˘}{𝔻}}}_{1}\times {\tilde{\overset{˘}{𝔻}}}_{2}\times {\tilde{\overset{˘}{𝔻}}}_{3}\times ...\times {\tilde{\overset{˘}{𝔻}}}_{n}$, and ${𝔓}^{\overset{˘}{𝔻}}$ denotes all the subsets of $\overset{˘}{𝔻}.$ An example of a HSS is tabulated in* Table 1.

**Table 1:** An example of a HSS in tabular form.

$\hat{¥}$	${\overset{˘}{w}}_{1}$	${\overset{˘}{w}}_{2}$	${\overset{˘}{w}}_{3}$	${\overset{˘}{w}}_{4}$	${\overset{˘}{w}}_{5}$	${\overset{˘}{w}}_{6}$	${\overset{˘}{w}}_{7}$	${\overset{˘}{w}}_{8}$
$\hbar_{1}$	1	0	1	0	1	0	1	1
$\hbar_{3}$	0	1	0	1	0	1	0	0
$\hbar_{5}$	1	1	0	0	1	1	0	0
$\hbar_{6}$	1	0	0	1	0	0	1	1
$\hbar_{9}$	1	1	0	1	1	0	1	1
$\hbar_{11}$	1	0	1	1	0	0	1	1
$\hbar_{14}$	0	0	1	1	1	1	0	1

**Example 3.1.** *Let $\text{Ł}=\left\{{\overset{˘}{w}}_{1},{\overset{˘}{w}}_{2},{\overset{˘}{w}}_{3},{\overset{˘}{w}}_{4},{\overset{˘}{w}}_{5},{\overset{˘}{w}}_{6},{\overset{˘}{w}}_{7},{\overset{˘}{w}}_{8}\right\}$, be the set of cars under consideration and let $\ddot{Y}=\{\alpha_{1},\alpha_{2},\alpha_{3},\alpha_{4}\}$ be a collection of parameters. Every parameter consists of a word or sentence, such that*

- ${\hat{\alpha }}_{1}$ *represents the parameter* **Performance**
- ${\hat{\alpha }}_{2}$ *represents the parameter* **Safety**
- ${\hat{\alpha }}_{3}$ *represents the parameter* **Comfort and Convenience**
- ${\hat{\alpha }}_{4}$ *represents the parameter* **Technology**

**Fig 1:**
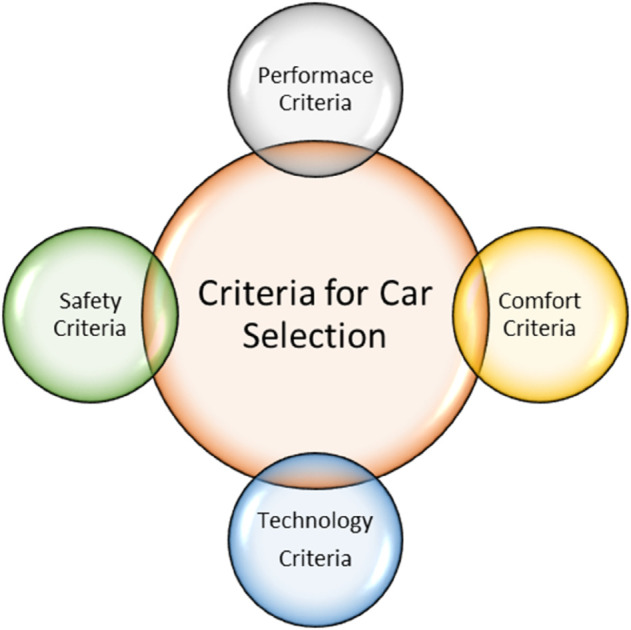
Major criteria for car selection.

**Fig 2:**
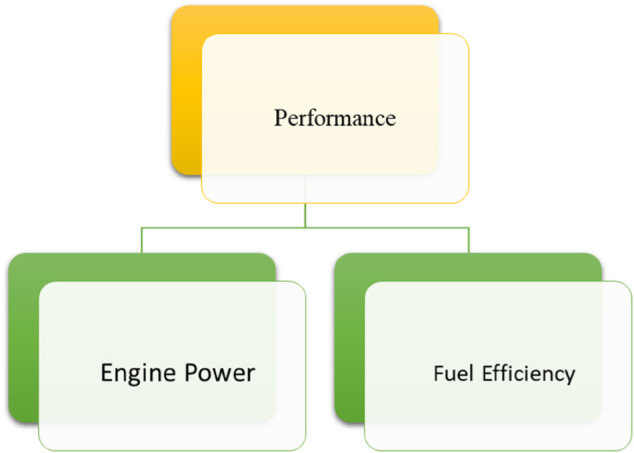
Sub-criteria of performance.

**Fig 3:**
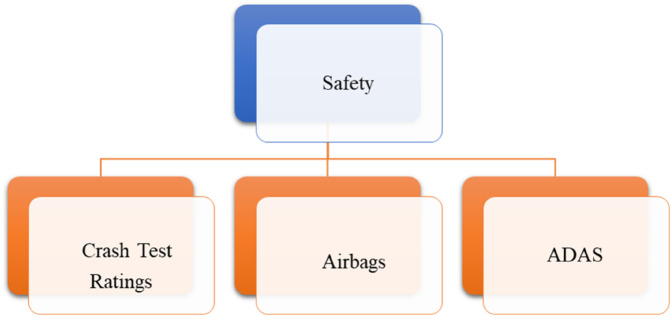
Sub-criteria of safety.

**Fig 4:**
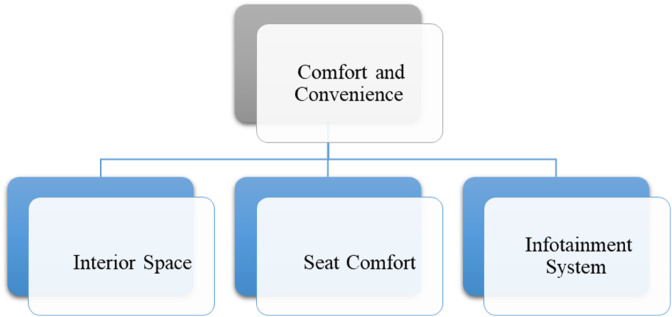
Sub-criteria of comfort and convenience.

*The disjoint sets consisting of sub values of attributes* $\alpha_{\hat{ı}}$ *are* ${\tilde{\overset{˘}{𝔻}}}_{1}=\{{\hat{\alpha }}_{11},{\hat{\alpha }}_{12}\}$, ${\tilde{\overset{˘}{𝔻}}}_{2}=\{{\hat{\alpha }}_{21},{\hat{\alpha }}_{22},{\hat{\alpha }}_{23}\}$, ${\tilde{\overset{˘}{𝔻}}}_{3}=\{{\hat{\alpha }}_{31},{\hat{\alpha }}_{32},{\hat{\alpha }}_{33}\}$, *and* ${\tilde{\overset{˘}{𝔻}}}_{4}=\{{\hat{\alpha }}_{41},{\hat{\alpha }}_{42},{\hat{\alpha }}_{43}\}$, *the cartesian product* ${\tilde{\overset{˘}{𝔻}}}_{1}\times {\tilde{\overset{˘}{𝔻}}}_{2}\times {\tilde{\overset{˘}{𝔻}}}_{3}\times {\tilde{\overset{˘}{𝔻}}}_{4}$ *will contain* 2×3×3×3=54 *elements. The selected parameters and the corresponding sub-parameter values are tabulated in* Table 2 *and their pictorial forms are presented by* Figs 1, 2, 3, 4*, and* 5.

**Fig 5:**
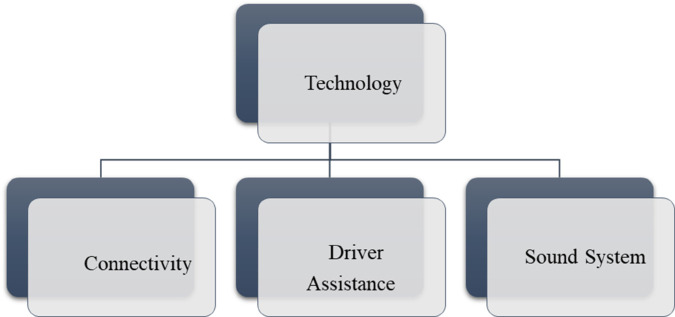
Sub-criteria of technology.

**Table 2:** The selected parameters and the corresponding sub-parameter values.

Parameters	Sub parametric values
${\hat{\alpha }}_{1}=$ Performance	${\hat{\alpha }}_{11}=$ Engine Power, ${\hat{\alpha }}_{12}=$ Fuel Efficiency,
${\hat{\alpha }}_{2}=$ Safety	${\hat{\alpha }}_{21}=$ Crash Test Ratings, ${\hat{\alpha }}_{22}=$ Airbags, ${\hat{\alpha }}_{23}=$ Advanced Driver Assistance Systems(ADAS),
${\hat{\alpha }}_{3}=$ Comfort and Convenience	${\hat{\alpha }}_{31}=$ Interior Space, ${\hat{\alpha }}_{32}=$ Seat Comfort, ${\hat{\alpha }}_{33}=$ Infotainment System
${\hat{\alpha }}_{4}=$ Technology	${\hat{a}}_{41}=$ Connectivity, ${\hat{\alpha }}_{42}=$ Driver Assistance Features, ${\hat{a}}_{43}=$ Sound System

*It would be impossible to describe all the possible combinations here, but this shows how to build the Cartesian product of these four sets.* $\overset{˘}{𝔻}={\tilde{\overset{˘}{𝔻}}}_{1}\times {\tilde{\overset{˘}{𝔻}}}_{2}\times {\tilde{\overset{˘}{𝔻}}}_{3},\times {\tilde{\overset{˘}{𝔻}}}_{4}=$


$$\left\{\begin{array}{c} \hbar_{1}=\left({\hat{\alpha }}_{11},{\hat{\alpha }}_{21},{\hat{\alpha }}_{31},{\hat{\alpha }}_{41}\right),\hbar_{2}=({\hat{\alpha }}_{11},{\hat{\alpha }}_{21},{\hat{\alpha }}_{32},{\hat{\alpha }}_{42}),\hbar_{3}=({\hat{\alpha }}_{11},{\hat{\alpha }}_{21},{\hat{\alpha }}_{31},{\hat{\alpha }}_{43}),\\ \hbar_{4}=({\hat{\alpha }}_{11},{\hat{\alpha }}_{21},{\hat{\alpha }}_{32},{\hat{\alpha }}_{41}),\hbar_{5}=({\hat{\alpha }}_{11},{\hat{\alpha }}_{21},{\hat{\alpha }}_{32},{\hat{\alpha }}_{42}),\;\hbar_{6}=({\hat{\alpha }}_{11},{\hat{\alpha }}_{21},{\hat{\alpha }}_{32},{\hat{\alpha }}_{43}),\\ \hbar_{7}=({\hat{\alpha }}_{11},{\hat{\alpha }}_{21},{\hat{\alpha }}_{33},{\hat{\alpha }}_{41}),\hbar_{8}=({\hat{\alpha }}_{11},{\hat{\alpha }}_{21},{\hat{\alpha }}_{33},{\hat{\alpha }}_{42}),\hbar_{9}=({\hat{\alpha }}_{11},{\hat{\alpha }}_{21},{\hat{\alpha }}_{33},{\hat{\alpha }}_{43}),\cdot \cdot \cdot ,\\ \hbar_{52}=({\hat{\alpha }}_{12},{\hat{\alpha }}_{22},{\hat{\alpha }}_{31},{\hat{\alpha }}_{42}),\hbar_{53}=({\hat{\alpha }}_{12},{\hat{\alpha }}_{22},{\hat{\alpha }}_{32},{\hat{\alpha }}_{41}),\hbar_{54}=({\hat{\alpha }}_{12},{\hat{\alpha }}_{22},{\hat{\alpha }}_{32},{\hat{\alpha }}_{44}) \end{array}\right\}$$

*Let* $\hat{𝕋}=\{\hbar_{1},\hbar_{3},\hbar_{5},\hbar_{6},\hbar_{9},\hbar_{11},\hbar_{14}\}\subseteq \overset{˘}{𝔻}$ *then the respective multi-argument approximate*


$$\begin{array}{c} \Lambda \left(\hbar_{1}\right)=\left\{{\overset{˘}{w}}_{1},{\overset{˘}{w}}_{3},{\overset{˘}{w}}_{5},{\overset{˘}{w}}_{7},{\overset{˘}{w}}_{8}\right\},\;\;\Lambda \left(\hbar_{3}\right)=\left\{{\overset{˘}{w}}_{2},{\overset{˘}{w}}_{4},{\overset{˘}{w}}_{6}\right\},\;\Lambda \left(\hbar_{5}\right)=\left\{{\overset{˘}{w}}_{1},{\overset{˘}{w}}_{2},{\overset{˘}{w}}_{5},{\overset{˘}{w}}_{6}\right\},\\ \;\Lambda \left(\hbar_{6}\right)=\left\{{\overset{˘}{w}}_{1},{\overset{˘}{w}}_{4},{\overset{˘}{w}}_{7},{\overset{˘}{w}}_{8}\right\},\Lambda \left(\hbar_{9}\right)=\{{\overset{˘}{w}}_{1},{\overset{˘}{w}}_{2},{\overset{˘}{w}}_{4},{\overset{˘}{w}}_{5},{\overset{˘}{w}}_{7},{\overset{˘}{w}}_{8}\},\;\Lambda \left(\hbar_{11}\right)=\left\{{\overset{˘}{w}}_{1},{\overset{˘}{w}}_{3},{\overset{˘}{w}}_{4},{\overset{˘}{w}}_{7},{\overset{˘}{w}}_{8}\right\},\\ \;\Lambda \left(\hbar_{14}\right)=\left\{{\overset{˘}{w}}_{3},{\overset{˘}{w}}_{4},{\overset{˘}{w}}_{5},{\overset{˘}{w}}_{6},{\overset{˘}{w}}_{8}\right\}.\; \end{array}$$

For any multi-argument tuple ℏ, the following function can be selected for a tabular representation of HSS $¥=(\Lambda ,\overset{˘}{𝔻})$


$$\hbar \left(\overset{˘}{\text{w}}\right)=\{\begin{array}{cc} 1 & \overset{˘}{\text{w}}\in \Lambda \left(\hbar \right)\\ 0 & \overset{˘}{\text{w}}\notin \Lambda \left(\hbar \right) \end{array}$$

Likewise, defining the pseudo HSS, a way to highlight the characteristics.

According to Definition 6, we get the following outcomes:


$$\begin{array}{c} \Lambda^{-1}({\overset{˘}{w}}_{1})=\{\hbar_{1},\hbar_{5},\hbar_{6},\hbar_{9},\hbar_{11}\},\;\;\Lambda^{-1}({\overset{˘}{w}}_{2})=\{\hbar_{3},\hbar_{5},\hbar_{9}\},\;\;\Lambda^{-1}({\overset{˘}{w}}_{3})=\{\hbar_{1},\hbar_{11},\hbar_{14}\},\;\\ \;\Lambda^{-1}({\overset{˘}{w}}_{4})=\{\hbar_{3},\hbar_{6},\hbar_{9},\hbar_{11},\hbar_{14}\},\;\;\Lambda^{-1}({\overset{˘}{w}}_{5})=\{\hbar_{1},\hbar_{5},\hbar_{9},\hbar_{14}\},\;\Lambda^{-1}({\overset{˘}{w}}_{6})=\{\hbar_{3},\hbar_{5},\hbar_{14}\},\;\\ \Lambda^{-1}({\overset{˘}{w}}_{7})=\{\hbar_{1},\hbar_{6},\hbar_{9},\hbar_{11}\},\Lambda^{-1}({\overset{˘}{w}}_{8})=\{\hbar_{1},\hbar_{6},\hbar_{9},\hbar_{11},\hbar_{14}\}. \end{array}$$

An example of a pseudo HSS is tabulated in Table 3.

**Table 3:** An example of a pseudo HSS in tabular form.

$\hat{¥}$	$\hbar_{1}$	$\hbar_{3}$	$\hbar_{5}$	$\hbar_{6}$	$\hbar_{9}$	$\hbar_{11}$	$\hbar_{14}$
${\overset{˘}{w}}_{1}$	1	0	1	1	1	1	0
${\overset{˘}{w}}_{2}$	0	1	1	0	1	0	0
${\overset{˘}{w}}_{3}$	1	0	0	0	0	1	1
${\overset{˘}{w}}_{4}$	0	1	0	1	1	1	1
${\overset{˘}{w}}_{5}$	1	0	1	0	1	0	1
${\overset{˘}{w}}_{6}$	0	1	1	0	0	0	1
${\overset{˘}{w}}_{7}$	1	0	0	1	1	1	0
${\overset{˘}{w}}_{8}$	1	0	0	1	1	1	1

**Definition 7.** *Consider Ł be a universal set and* $\ddot{Y}=\{{\hat{\alpha }}_{1},{\hat{\alpha }}_{2},{\hat{\alpha }}_{3},...,{\hat{\alpha }}_{n}\}$ *is a set of attributes. The corresponding attribute values for every attribute* ${\hat{\alpha }}_{\hat{ı}},\hat{ı}=1,2,3,...,n$ *are respectively the sets* ${\tilde{\overset{˘}{𝔻}}}_{1},{\tilde{\overset{˘}{𝔻}}}_{2},{\tilde{\overset{˘}{𝔻}}}_{3},...,{\tilde{\overset{˘}{𝔻}}}_{n}$, *with* ${\tilde{\overset{˘}{𝔻}}}_{\hat{ı}}\;\cap \;{\tilde{\overset{˘}{𝔻}}}_{\hat{j}}=\phi ,\;for\;\;\hat{ı}\neq \hat{j}$, *and* $\hat{ı},\hat{j}\in ℵ.$ *Then the pair* $\left({\tilde{\Lambda }}^{-1},\overset{˘}{𝔻}\right)$ *is called a pseudo fuzzy HSS over* Ł *iff* ${\tilde{\Lambda }}^{-1}:⟶{\hat{𝔽}}^{\overset{˘}{𝔻}}$, *where* $\overset{˘}{𝔻}={\tilde{\overset{˘}{𝔻}}}_{1}\times {\tilde{\overset{˘}{𝔻}}}_{2}\times {\tilde{\overset{˘}{𝔻}}}_{3}\times ...\times {\tilde{\overset{˘}{𝔻}}}_{n}$, *and* ${\hat{𝔽}}^{\overset{˘}{𝔻}}$ *denotes all the fuzzy subsets of* $\overset{˘}{𝔻}.$

**Example 3.2** *Consider Example 3.1 we have*


$$\begin{array}{c} \Lambda \left(\hbar_{1}\right)=\left\{\frac{{\overset{˘}{w}}_{1}}{0.7},\frac{{\overset{˘}{w}}_{3}}{0.3},\frac{{\overset{˘}{w}}_{5}}{0.2},\frac{{\overset{˘}{w}}_{7}}{0.1},\frac{{\overset{˘}{w}}_{8}}{0.6}\right\},\;\Lambda \left(\hbar_{3}\right)=\left\{\frac{{\overset{˘}{w}}_{2}}{0.4},\frac{{\overset{˘}{w}}_{4}}{0.3},\frac{{\overset{˘}{w}}_{6}}{0.8}\right\},\Lambda \left(\hbar_{5}\right)=\left\{\frac{{\overset{˘}{w}}_{1}}{0.9},\frac{{\overset{˘}{w}}_{2}}{0.3},\frac{{\overset{˘}{w}}_{5}}{0.1},\frac{{\overset{˘}{w}}_{6}}{0.7}\right\},\\ \Lambda \left(\hbar_{6}\right)=\left\{\frac{{\overset{˘}{w}}_{1}}{0.2},\frac{{\overset{˘}{w}}_{4}}{0.3},\frac{{\overset{˘}{w}}_{7}}{0.6},\frac{{\overset{˘}{w}}_{8}}{0.9}\right\},\Lambda \left(\hbar_{9}\right)=\left\{\frac{{\overset{˘}{w}}_{1}}{0.3},\frac{{\overset{˘}{w}}_{2}}{0.5},\frac{{\overset{˘}{w}}_{4}}{0.2},\frac{{\overset{˘}{w}}_{5}}{0.5},\frac{{\overset{˘}{w}}_{7}}{0.7},\frac{{\overset{˘}{w}}_{8}}{0.4}\right\},\\ \Lambda \left(\hbar_{11}\right)=\left\{\frac{{\overset{˘}{w}}_{1}}{0.4},\frac{{\overset{˘}{w}}_{3}}{0.2},\frac{{\overset{˘}{w}}_{4}}{0.3},\frac{{\overset{˘}{w}}_{7}}{0.5},\frac{{\overset{˘}{w}}_{8}}{0.7}\right\},\Lambda \left(\hbar_{14}\right)=\left\{\frac{{\overset{˘}{w}}_{3}}{0.5},\frac{{\overset{˘}{w}}_{4}}{0.3},\frac{{\overset{˘}{w}}_{5}}{0.6},\frac{{\overset{˘}{w}}_{6}}{0.2},\frac{{\overset{˘}{w}}_{8}}{0.1}\right\},\; \end{array}$$

*An example of fuzzy HSS is tabulated in* Table 4.

**Table 4:** An example of fuzzy HSS in tabular form.

$\hat{¥}$	${\overset{˘}{w}}_{1}$	${\overset{˘}{w}}_{2}$	${\overset{˘}{w}}_{3}$	${\overset{˘}{w}}_{4}$	${\overset{˘}{w}}_{5}$	${\overset{˘}{w}}_{6}$	${\overset{˘}{w}}_{7}$	${\overset{˘}{w}}_{8}$
$\hbar_{1}$	0.7	0	0.3	0	0.2	0	0.1	0.6
$\hbar_{3}$	0	0.4	0	0.3	0	0.8	0	0
$\hbar_{5}$	0.9	0.3	0	0	0.1	0.7	0	0
$\hbar_{6}$	0.2	0	0	0.3	0	0	0.6	0.9
$\hbar_{9}$	0.3	0.5	0	0.2	0.5	0	0.7	0.4
$\hbar_{11}$	0.4	0	0.2	0.3	0	0	0.5	0.7
$\hbar_{14}$	0	0	0.5	0.3	0.6	0.2	0	0.1

*Likewise, defining the pseudo HSS, a way to highlight the characteristics.*

According to Definition 7, we get the following outcomes:


$$\begin{array}{c} \Lambda^{-1}({\overset{˘}{w}}_{1})=\{\frac{\hbar_{1}}{0.3},\frac{\hbar_{5}}{0.2},\frac{\hbar_{6}}{0.5},\frac{\hbar_{9}}{0.1},\frac{\hbar_{11}}{0.4}\},\;\;\;\Lambda^{-1}({\overset{˘}{w}}_{2})=\{\frac{\hbar_{3}}{0.2},\frac{\hbar_{5}}{0.8},\frac{\hbar_{9}}{0.7}\},\;\;\;\Lambda^{-1}({\overset{˘}{w}}_{3})=\{\frac{\hbar_{1}}{0.7},\frac{\hbar_{11}}{0.5},\frac{\hbar_{14}}{0.3}\},\;\\ \;\Lambda^{-1}({\overset{˘}{w}}_{4})=\{\frac{\hbar_{3}}{0.4},\frac{\hbar_{6}}{0.5},\frac{\hbar_{9}}{0.3},\frac{\hbar_{11}}{0.8},\frac{\hbar_{14}}{0.1}\},\;\;\;\Lambda^{-1}({\overset{˘}{w}}_{5})=\{\frac{\hbar_{1}}{0.2},\frac{\hbar_{5}}{0.9},\frac{\hbar_{9}}{0.5},\frac{\hbar_{14}}{0.7}\},\;\Lambda^{-1}({\overset{˘}{w}}_{6})=\{\frac{\hbar_{3}}{0.9},\frac{\hbar_{5}}{0.3},\frac{\hbar_{14}}{0.4}\},\;\\ \Lambda^{-1}({\overset{˘}{w}}_{7})=\{\frac{\hbar_{1}}{0.5},\frac{\hbar_{6}}{0.1},\frac{\hbar_{9}}{0.7},\frac{\hbar_{11}}{0.2}\},\;\Lambda^{-1}({\overset{˘}{w}}_{8})=\{\frac{\hbar_{1}}{0.4},\frac{\hbar_{6}}{0.3},\frac{\hbar_{9}}{0.6},\frac{\hbar_{11}}{0.8},\frac{\hbar_{14}}{0.5}\}. \end{array}$$

An example of a pseudo fuzzy HSS is tabulated in Table 5.

**Table 5:** An example of a pseudo fuzzy HSS in tabular form.

$\hat{¥}$	$\hbar_{1}$	$\hbar_{3}$	$\hbar_{5}$	$\hbar_{6}$	$\hbar_{9}$	$\hbar_{11}$	$\hbar_{14}$
${\overset{˘}{w}}_{1}$	0.3	0	0.2	0.5	0.1	0.4	0
${\overset{˘}{w}}_{2}$	0	0.2	0.8	0	0.7	0	0
${\overset{˘}{w}}_{3}$	0.7	0	0	0	0	0.5	0.5
${\overset{˘}{w}}_{4}$	0	0.4	0	0.5	0.3	0.8	0.1
${\overset{˘}{w}}_{5}$	0.2	0	0.9	0	0.5	0	0.7
${\overset{˘}{w}}_{6}$	0	0.9	0.3	0	0	0	0.4
${\overset{˘}{w}}_{7}$	0.5	0	0	0.1	0.7	0.2	0
${\overset{˘}{w}}_{8}$	0.4	0	0	0.3	0.6	0.8	0.5

**Definition 8.** *Consider a pseudo fuzzy HSS $¥=({\tilde{\Lambda }}^{-1},\overset{˘}{𝔻})$ over Ł. We named $\hat{T}=(\text{Ł},¥)$ the hypersoft fuzzy approximation space with $\hbar_{\hat{ı}}\in \overset{˘}{𝔻}$ a $\hat{𝔫}-$ argument tuple. For any $𝕁\in {\hat{𝔽}}^{\overset{˘}{𝔻}}$, we define $\overset{\leftarrow }{\Omega }(𝕁)$ and $\vec{\Omega }(𝕁)$ with respect to the hypersoft fuzzy approximation space $\hat{T}=(\text{Ł},¥)$ are the fuzzy sets of Ł and their membership functions for each ℓ∈Ł, defined by*


$$\overset{\leftarrow }{\Omega }(𝕁)(\ell )={⋀}_{℘\in \overset{˘}{𝔻}}\left[\left((1-{\tilde{\Lambda }}^{-1}(\ell )(℘))\right)\vee (𝕁)(℘)\right],\;\ell \in \text{Ł},$$

$$\vec{\Omega }(𝕁)(\ell )={⋁}_{℘\in \overset{˘}{𝔻}}\left[\Lambda^{-1}(\ell )(℘)\wedge (𝕁)(℘)\right],\;\ell \in \text{Ł}.$$

*The sets $\overset{\leftarrow }{\Omega }(𝕁)$ and $\vec{\Omega }(𝕁)$ known as $\hat{T}-$ lower hypersoft fuzzy approximation and $\hat{T}-$ upper hypersoft fuzzy approximation respectively. We call the pair $\left(\overset{\leftarrow }{\Omega }(𝕁),\vec{\Omega }(𝕁)\right)$ a hypersoft fuzzy rough set.*

**Example 3.3** *Given a subset $𝕁\in {\hat{𝔽}}^{\overset{˘}{𝔻}}.$ The values of the membership as:*


$$𝕁=\frac{0.7}{\hbar_{1}}+\frac{0.5}{\hbar_{3}}+\frac{0.4}{\hbar_{5}}+\frac{0.2}{\hbar_{6}}+\frac{0.1}{\hbar_{9}}+\frac{0.8}{\hbar_{11}}+\frac{0.3}{\hbar_{14}}.$$

*by Definition 8, the lower and upper approximation of 𝕁, respectively, as follows*


$$\begin{array}{c} \overset{\leftarrow }{\Omega }(𝕁)(\overset{˘}{w})={⋀}_{\hbar \in \overset{˘}{𝔻}}\left[\left((1-{\tilde{\Lambda }}^{-1}(\overset{˘}{w})(\hbar ))\right)\vee (𝕁)(\hbar )\right],\;\overset{˘}{w}\in \text{Ł} \end{array}$$

$$\begin{array}{c} \vec{\Omega }(𝕁)(\overset{˘}{w})={⋁}_{\hbar \in \overset{˘}{𝔻}}\left[{\tilde{\Lambda }}^{-1}(\overset{˘}{w})(\hbar )\wedge (𝕁)(\hbar )\right],\;\overset{˘}{w}\in \text{Ł}. \end{array}$$

Table 5 *allows us to obtain the fuzzy lower and upper approximations of 𝕁 in the following way:*


$$\begin{array}{c} \overset{\leftarrow }{\Omega }\left(𝕁\right)\left({\overset{˘}{w}}_{1}\right)=0.5,\;\vec{\Omega }\left(𝕁\right)\left({\overset{˘}{w}}_{1}\right)=0.4,\;\overset{\leftarrow }{\Omega }\left(𝕁\right)\left({\overset{˘}{w}}_{2}\right)=0.3,\;\vec{\Omega }\left(𝕁\right)\left({\overset{˘}{w}}_{2}\right)=0.4,\\ \overset{\leftarrow }{\Omega }\left(𝕁\right)\left({\overset{˘}{w}}_{3}\right)=0.5,\;\vec{\Omega }\left(𝕁\right)\left({\overset{˘}{w}}_{3}\right)=0.5,\;\overset{\leftarrow }{\Omega }\left(𝕁\right)\left({\overset{˘}{w}}_{4}\right)=0.5,\;\vec{\Omega }\left(𝕁\right)\left({\overset{˘}{w}}_{4}\right)=0.8,\\ \overset{\leftarrow }{\Omega }\left(𝕁\right)\left({\overset{˘}{w}}_{5}\right)=0.5,\;\vec{\Omega }\left(𝕁\right)\left({\overset{˘}{w}}_{5}\right)=0.4,\;\overset{\leftarrow }{\Omega }\left(𝕁\right)\left({\overset{˘}{w}}_{6}\right)=0.5,\;\vec{\Omega }\left(𝕁\right)\left({\overset{˘}{w}}_{6}\right)=0.5,\\ \overset{\leftarrow }{\Omega }\left(𝕁\right)\left({\overset{˘}{w}}_{7}\right)=0.3,\;\vec{\Omega }\left(𝕁\right)\left({\overset{˘}{w}}_{7}\right)=0.5,\;\overset{\leftarrow }{\Omega }\left(𝕁\right)\left({\overset{˘}{w}}_{8}\right)=0.4,\;\vec{\Omega }\left(𝕁\right)\left({\overset{˘}{w}}_{8}\right)=0.8. \end{array}$$

*In other words, for each parameter set* $\overset{˘}{𝔻}$, *we get the upper and lower approximations of the fuzzy subset* 𝕁.


$$\overset{\leftarrow }{\Omega }\left(𝕁\right)=\frac{0.5}{{\overset{˘}{w}}_{1}}+\frac{0.3}{{\overset{˘}{w}}_{2}}+\frac{0.5}{{\overset{˘}{w}}_{3}}+\frac{0.5}{{\overset{˘}{w}}_{4}}+\frac{0.5}{{\overset{˘}{w}}_{5}}+\frac{0.5}{{\overset{˘}{w}}_{6}}+\frac{0.3}{{\overset{˘}{w}}_{7}}+\frac{0.4}{{\overset{˘}{w}}_{8}},$$

$$\vec{\Omega }\left(𝕁\right)=\frac{0.4}{{\overset{˘}{w}}_{1}}+\frac{0.4}{{\overset{˘}{w}}_{2}}+\frac{0.5}{{\overset{˘}{w}}_{3}}+\frac{0.8}{{\overset{˘}{w}}_{4}}+\frac{0.4}{{\overset{˘}{w}}_{5}}+\frac{0.5}{{\overset{˘}{w}}_{6}}+\frac{0.5}{{\overset{˘}{w}}_{7}}+\frac{0.8}{{\overset{˘}{w}}_{8}}.$$

*Evidently* $\overset{\leftarrow }{\Omega }\left(𝕁\right)⊈\vec{\Omega }\left(𝕁\right).$

**Proposition 1.** *Let* $\hat{T}=(\text{Ł},¥)$ *be hypersoft fuzzy approximation space. Given* $𝕁\in {\hat{𝔽}}^{\overset{˘}{𝔻}}$, *we have*

*1.* $\overset{\leftarrow }{\Omega }\left(𝕁\right)=\sim \vec{\Omega }\left(\sim 𝕁\right)$,
*2.* $\vec{\Omega }\left(𝕁\right)=\sim \overset{\leftarrow }{\Omega }\left(\sim 𝕁\right).$

*according to Proposition 1 the hypersoft fuzzy rough approximation operators $\overset{\leftarrow }{\Omega }$ and $\vec{\Omega }$ are dual to each other. In addition, the following findings are evident for this operators.*

**Theorem 9.** *Consider $\hat{T}=(\text{Ł},¥)$ be the hypersoft fuzzy approximation space. For any $𝕁,𝕐\in {\hat{𝔽}}^{\overset{˘}{𝔻}}$, we have*

*1.* $\overset{\leftarrow }{\Omega }\left(𝕁\cap 𝕐\right)=\overset{\leftarrow }{\Omega }\left(𝕁\right)\cap \overset{\leftarrow }{\Omega }\left(𝕁\right).$
*2.* $\vec{\Omega }\left(𝕁\cup 𝕐\right)=\vec{\Omega }\left(𝕁\right)\cup \vec{\Omega }\left(𝕁\right).$
*3.* $\overset{\leftarrow }{\Omega }\left(𝕁\cup 𝕐\right)⊇\overset{\leftarrow }{\Omega }\left(𝕁\right)\cup \overset{\leftarrow }{\Omega }\left(𝕁\right).$
*4.* $\vec{\Omega }\left(𝕁\cap 𝕐\right)\subseteq \vec{\Omega }\left(𝕁\right)\cap \vec{\Omega }\left(𝕁\right).$
*5.* $𝕁\subseteq 𝕐⟶\overset{\leftarrow }{\Omega }\left(𝕁\right)\subseteq \overset{\leftarrow }{\Omega }\left(𝕐\right).$
*6.* $\vec{\Omega }\left(𝕁\right)\subseteq \vec{\Omega }\left(𝕐\right).$

*With the help of Definition 8 and Example 3.3, the results above are readily displayed.*

**Remark 1.** *Assume that $¥=({\tilde{\Lambda }}^{-1},\overset{˘}{𝔻})$ be a pseudo HSS over Ł, we call $\hat{T}=(\text{Ł},¥)$ the hypersoft appeoximation space, and the following forms are what the soft fuzzy rough approximation operators mentioned above summarize as:*


$$\overset{\leftarrow }{\Omega }(𝕁)(\ell )={⋀}_{℘\in {\tilde{\Lambda }}^{-1}(\ell )}(𝕁)(℘),\;\ell \in \text{Ł},$$

$$\vec{\Omega }(𝕁)(\ell )={⋁}_{℘\in {\tilde{\Lambda }}^{-1}(\ell )}(𝕁)(℘),\;\ell \in \text{Ł}.$$

*Here, we refer the pair $\left(\overset{\leftarrow }{\Omega }(𝕁),\vec{\Omega }(𝕁)\right)$ a hypersoft rough fuzzy set. Which is the specific case of hypersoft fuzzy rough set if we apply the condition of pseudo mapping* ${\tilde{\Lambda }}^{-1}.$

**Remark 2.** *Assume that $\hat{T}=(\text{Ł},¥)$ be the hypersoft fuzzy approximation space, and $𝕁\in {𝔓}^{\overset{˘}{𝔻}}$. Then the following are the hypersoft fuzzy rough approximation operators:*


$$\overset{\leftarrow }{\Omega }(𝕁)(\ell )={⋀}_{℘\notin 𝕁}\left[\left(1-{\tilde{\Lambda }}^{-1}(\ell )(℘)\right)\right],\;\ell \in \text{Ł},$$

$$\vec{\Omega }(𝕁)(\ell )={⋁}_{℘\in 𝕁}\left[\Lambda^{-1}(\ell )(℘)\right],\;\ell \in \text{Ł}.$$

*Where $\overset{\leftarrow }{\Omega }(𝕁)$ and $\vec{\Omega }(𝕁)$ shows the approximation of any crisp subset in $\overset{˘}{𝔻}$ on the hypersoft fuzzy approximation space $\hat{T}=(\text{Ł},¥).$*

## 4 Industrial fault diagnosis using hypersoft fuzzy rough approximations

Because a manufacturing plant uses a variety of machinery and equipment on a constant basis, wear and tear, poor maintenance, and unforeseen circumstances can all lead to malfunctions. To prevent downtime and guarantee seamless operations, early and precise detection of these issues is essential. But the information gleaned from sensors and logs might be inaccurate or lacking. In these circumstances, rough hypersoft sets can be useful in managing the ambiguity and uncertainty in the data.

In industrial fault diagnosis, the application of HRS stands out due to the distinct demands and challenges of diagnosing faults accurately under uncertain and complex conditions. Unlike general multi-criteria decision-making domains, fault diagnosis in industrial settings often requires handling vast amounts of imprecise, overlapping, and dynamic data. HRS provides a unique capability to deal with these uncertainties by accommodating multi-criteria assessments within high-dimensional, layered data structures. Specifically, HRS facilitates a finer granularity in capturing interdependencies between criteria that may evolve or conflict due to fluctuating operating conditions. This characteristic is particularly valuable in industrial contexts where fault sources can vary in impact and frequency. Compared to other domains, industrial fault diagnosis with HRS offers a more nuanced approach to processing uncertainty and prioritizing fault indicators, allowing for adaptable and context-sensitive fault detection, which is critical for maintaining operational efficiency and safety.

### 4.1 Modified Bingzhen and Weimin’s decision model

The following decision model is the modified version of decision model discussed by Bingzhen and Weimin [49]. Assume that $\text{Ł}=\left\{{\overset{˘}{w}}_{1},{\overset{˘}{w}}_{2},{\overset{˘}{w}}_{3},...,{\overset{˘}{w}}_{𝕜}\right\}$, be the 𝕜 number of fault diagnosis. ${\tilde{\Lambda }}^{-1}\in \Omega (\text{Ł}\;\times \;\overset{˘}{𝔻})$ is the fuzzy mapping in the fault diagnosis set Ł to $\overset{˘}{𝔻}$. That is ${\tilde{\Lambda }}^{-1}\left(\ell_{\tilde{\iota }}\right)\left(\hbar_{\hat{ı}}\right)\in [0,1]$ where $\forall (\ell_{\tilde{\iota }}\in \text{Ł},\hbar_{\hat{ı}}\in \overset{˘}{𝔻}).$ Thus we construct a hypersoft fuzzy information system $\hat{T}=(\text{Ł},¥)$ for the evaluation of fault diagnosis. Then we have


$${𝕁}^{+}=\sum \frac{\max\tilde{\Lambda }\left(\hbar_{\hat{ı}}\right)}{\hbar_{\hat{ı}}},\;\forall \;\hat{ı}\in \overset{˘}{𝔻},$$

$${𝕁}^{+}=max\left\{\tilde{\Lambda }\left(\hbar_{\hat{ı}}\right)\left(\ell_{\tilde{\iota }}\right)|\ell_{\tilde{\iota }}\in \text{Ł}\right\},$$ (8)

$${𝕁}^{-}=\sum \frac{\min\tilde{\Lambda }\left(\hbar_{\hat{ı}}\right)}{\hbar_{\hat{ı}}},\;\forall \;\hat{ı}\in \overset{˘}{𝔻},$$

$${𝕁}^{-}=min\left\{\tilde{\Lambda }\left(\hbar_{\hat{ı}}\right)\left(\ell_{\tilde{\iota }}\right)|\ell_{\tilde{\iota }}\in \text{Ł}\right\}.$$ (9)

By calculating the maximum and minimum values for the fuzzy HSS $\left(\tilde{\Lambda },\overset{˘}{𝔻}\right)$ concerning the characteristic factor $\forall \;\hbar_{\hat{ı}}\in \overset{˘}{𝔻}.$, it is simple to determine that ${𝕁}^{+}$ and ${𝕁}^{-}$ are established. Furthermore, there is ${𝕁}^{+},{𝕁}^{-}\in {\hat{𝔽}}^{\overset{˘}{𝔻}}.$ The steps of the evaluation model are described in detail below. Initially, we compute the object’s lower approximation $\overset{\leftarrow }{\Omega }\left({𝕁}^{+}\right)\overset{\leftarrow }{\Omega }\left({𝕁}^{-}\right)$ and upper approximation $\vec{\Omega }\left({𝕁}^{+}\right)\vec{\Omega }\left({𝕁}^{-}\right)$ with respect to the hypersoft fuzzy information system $\hat{T}=(\text{Ł},¥)$ respectively, as follows,


$$\overset{\leftarrow }{\Omega }({𝕁}^{+})(\ell_{\hat{\iota }})={⋀}_{\hbar_{\hat{j}}\in \overset{˘}{𝔻}}\left[\left(1-{\tilde{\Lambda }}^{-1}(\ell_{\hat{\iota }})(\hbar_{\hat{j}})\right)\vee {𝕁}^{+}(\hbar_{\hat{j}})\right],\;\ell_{\hat{\iota }}\in \text{Ł},$$

$$\vec{\Omega }({𝕁}^{+})(\ell_{\hat{\iota }})={⋁}_{\hbar_{\hat{j}}\in \overset{˘}{𝔻}}\left[{\tilde{\Lambda }}^{-1}(\ell_{\hat{\iota }})(\hbar_{\hat{j}})\wedge {𝕁}^{-}(\hbar_{\hat{j}})\right],\;\ell_{\hat{\iota }}\in \text{Ł},$$

and


$$\overset{\leftarrow }{\Omega }({𝕁}^{-})(\ell_{\hat{\iota }})={⋀}_{\hbar_{\hat{j}}\in \overset{˘}{𝔻}}\left[\left(1-{\tilde{\Lambda }}^{-1}(\ell_{\hat{\iota }})(\hbar_{\hat{j}})\right)\vee {𝕁}^{-}(\hbar_{\hat{j}})\right],\;\ell_{\hat{\iota }}\in \text{Ł},$$

$$\vec{\Omega }({𝕁}^{-})(\ell_{\hat{\iota }})={⋁}_{\hbar_{\hat{j}}\in \overset{˘}{𝔻}}\left[{\tilde{\Lambda }}^{-1}(\ell_{\hat{\iota }})(\hbar_{\hat{j}})\wedge {𝕁}^{-}(\hbar_{\hat{j}})\right],\;\ell_{\hat{\iota }}\in \text{Ł},$$

where $\hat{\iota }=1,2,3,...,𝔪,\;\hat{j}=1,2,3,...,𝔫.$

We describe the following ideas using the fundamental theory of hypersoft sets.

**Definition 10.** *Assume that $\hat{T}=(\text{Ł},¥)$ be the hypersoft fuzzy approximation space. Whenever $𝕁\in {\hat{𝔽}}^{\overset{˘}{𝔻}}$, we call*

$$\xi \left(𝕁\right)=\overset{\leftarrow }{\Omega }\left(𝕁\right)\left(\tilde{x}\right)+\vec{\Omega }\left(𝕁\right)\left(\tilde{x}\right),\;\tilde{x}\in \text{Ł}$$ (10)

*the 𝕁 score function in relation to the hypersoft fuzzy approximation space.*

Second, we determine the score value for every fault diagnosis $\ell_{\hat{\iota }}\in \text{Ł}$ concerning ${𝕁}^{+}$ and ${𝕁}^{-}$ as follows, respectively, using Definition 10.


$$\xi_{\hat{ı}}\left({𝕁}^{+}\right)=\overset{\leftarrow }{\Omega }\left({𝕁}^{+}\right)\left(\ell_{\hat{\iota }}\right)+\vec{\Omega }\left({𝕁}^{+}\right)\left(\ell_{\hat{\iota }}\right),\;\ell_{\hat{\iota }}\in \text{Ł}$$

$$\xi_{\hat{ı}}\left({𝕁}^{-}\right)=\overset{\leftarrow }{\Omega }\left({𝕁}^{-}\right)\left(\ell_{\hat{\iota }}\right)+\vec{\Omega }\left({𝕁}^{-}\right)\left(\ell_{\hat{\iota }}\right),\;\ell_{\hat{\iota }}\in \text{Ł}$$

We introduce the notion of hypersoft close degree over the hypersoft fuzzy approximation space in the following.

**Definition 11.** *Assume that $\hat{T}=(\text{Ł},¥)$ be a hypersoft fuzzy information system for evaluation of the fault diagnosis. For the object ${𝕁}^{+}$ and ${𝕁}^{-}$, we call*


$$\xi_{\hat{ı}}=\xi_{\hat{ı}}\left({𝕁}^{+}\right)-\xi_{\hat{ı}}\left({𝕁}^{-}\right),$$

*the hypersoft close degree of the $\hat{\iota }th$ fault diagnosis plan $\ell_{\tilde{\iota }}$ about $\hat{T}=(\text{Ł},¥).$*

**Remark 3.** *Based on the characteristics of the hypersoft fuzzy rough set, if* ${𝕁}_{1},{𝕁}_{2}\in {\hat{𝔽}}^{\overset{˘}{𝔻}}$, *which satisfies* ${𝕁}_{1}\subseteq {𝕁}_{2},\;\overset{\leftarrow }{\Omega }\left({𝕁}_{1}\right)\subseteq \overset{\leftarrow }{\Omega }\left({𝕁}_{2}\right)$ *and* $\vec{\Omega }\left({𝕁}_{1}\right)\subseteq \vec{\Omega }\left({𝕁}_{2}\right)$ *hold. It is evident that* ${𝕁}^{+}$ *and* ${𝕁}^{-}$ *satisfy* ${𝕁}^{-}\subseteq {𝕁}^{+}$ thus $\xi_{\hat{ı}}\left({𝕁}^{+}\right)\geq \xi_{\hat{ı}}\left({𝕁}^{-}\right)$ *for any* $\ell_{\tilde{\iota }}\in \text{Ł}.$ *So* $\xi_{\hat{ı}}\geq 0.$

Ultimately, based on the values of the hypersoft close degree, we are able to provide an extensive assessment and rating of all the fault diagnosis strategies for a certain fault event.

### 4.2 Problem statement:

Conveyor belt systems are essential parts of several industries in Pakistan, such as manufacturing, logistics, mining, and agriculture. These systems are essential to the smooth operation of manufacturing lines and supply chains because they make the efficient movement of materials and goods possible. Conveyor belts can, however, develop a variety of problems that can cause serious operational disruptions, higher maintenance expenses, and safety issues. For Pakistani conveyor belt systems to be dependable and efficient, effective fault identification is necessary. Although these systems are effective at transporting substances, wear, misalignment, contamination, and other variables can cause malfunctions and failures. The main goal is to create a thorough and efficient diagnostic technique for identifying and categorizing conveyor belt system flaws in Pakistani companies. Numerous factors, including vibration level, temperature, belt tension, motor current, and noise level, should be incorporated into the diagnosis process. To reduce system downtime and maintenance costs, the goal is to precisely identify defects, anticipate possible breakdowns, and offer practical repair recommendations.

## 5 Adopted parameters and their roles description

In this section, the evaluating parameters and their related sub-parameters are discussed. The parameters and their relevant sub-parameters are taken from the literature [53–55]. In general, the conveyor belt should have the following characteristics:

(1). Vibration Level $\left({\hat{\alpha }}_{1}\right)$
  1.1. ${\hat{\alpha }}_{11}$= Misalignment: Uneven vibration can occur when conveyor belt system components are not properly aligned. Frequently, periodic increases in vibration amplitude are indicative of this.
  1.2. ${\hat{\alpha }}_{12}$= Unbalanced Load: Uneven distribution of the conveyor belt’s load may cause irregular and heightened vibration patterns.
  1.3. ${\hat{\alpha }}_{13}$= Mechanical Wear: Uneven vibrations can be caused by worn-out parts like pulleys or rollers.
  1.4. ${\hat{\alpha }}_{14}$= Bearing Failures: For the conveyor system to run smoothly, bearings are essential. Significant increases in vibration might result from any wear or flaws in the bearings.
(2). Temperature $\left({\hat{\alpha }}_{2}\right)$
  2.1. ${\hat{\alpha }}_{21}$= Overheating Motors: Internal malfunctions, overload, and inadequate ventilation can all cause motors to overheat. High temperatures close to the motor are a sign of this issue.
  2.2. ${\hat{\alpha }}_{22}$= Lubrication Problems: Moving parts may generate more heat and friction if their lubrication is inadequate or deteriorated.
  2.3. ${\hat{\alpha }}_{23}$= Excessive Friction: Increased friction can be caused by misalignment, tension problems, or worn-out parts, which raises the temperature.
  2.4. ${\hat{\alpha }}_{24}$= Bearing Failures: Unusual heat buildup might result from worn or defective bearings because of increased wear and friction.
(3). Belt Tension $\left({\hat{\alpha }}_{3}\right)$
  3.1. ${\hat{\alpha }}_{31}$= Misalignment: Unusual stress levels can result from uneven tension across the belt caused by misaligned pulleys or rollers.
  3.2. ${\hat{\alpha }}_{32}$= Improper Loading: Variations in belt tension due to uneven or excessive loading may result in slippage or strain.
  3.3. ${\hat{\alpha }}_{33}$= Wear and Tear: Belt deterioration or stretching over time might result in uneven tension levels.
  3.4. ${\hat{\alpha }}_{34}$= Mechanical Issues: Inadequate belt tension might result from issues with mechanical parts or tensioning mechanisms.
(4). Motor Current $\left({\hat{\alpha }}_{4}\right)$
  4.1. ${\hat{\alpha }}_{41}$= Overload: The motor may draw more current than usual if there is an excessive material load or mechanical resistance.
  4.2. ${\hat{\alpha }}_{42}$= Electrical Faults: Anomalies involving wiring, insulation problems, or short circuits can result in anomalous current levels.
  4.3. ${\hat{\alpha }}_{43}$= Mechanical Problems: Due to increased friction or resistance, worn-out or damaged mechanical parts, such as gears or bearings, can raise the motor’s current draw.
  4.4. ${\hat{\alpha }}_{44}$= Misalignment: Component misalignment may result in increased mechanical resistance, increasing motor load and current consumption.
(5). Noise Level $\left({\hat{\alpha }}_{5}\right)$
  5.1. ${\hat{\alpha }}_{51}$= Mechanical Wear: Unusual noise can be produced by worn-out parts like rollers or bearings because of increased friction or looseness.
  5.2. ${\hat{\alpha }}_{52}$= Misalignment: When a belt travels, misaligned pulleys or rollers can cause uneven contact and noise.
  5.3. ${\hat{\alpha }}_{53}$= Bearing Failures: Because of increased friction and vibration, defective or broken bearings can produce noise.
  5.4. ${\hat{\alpha }}_{54}$= Loose or Damaged Components: When the conveyor is operating, loose or broken pieces may rattle or make strange noises.

**Fig 6:**
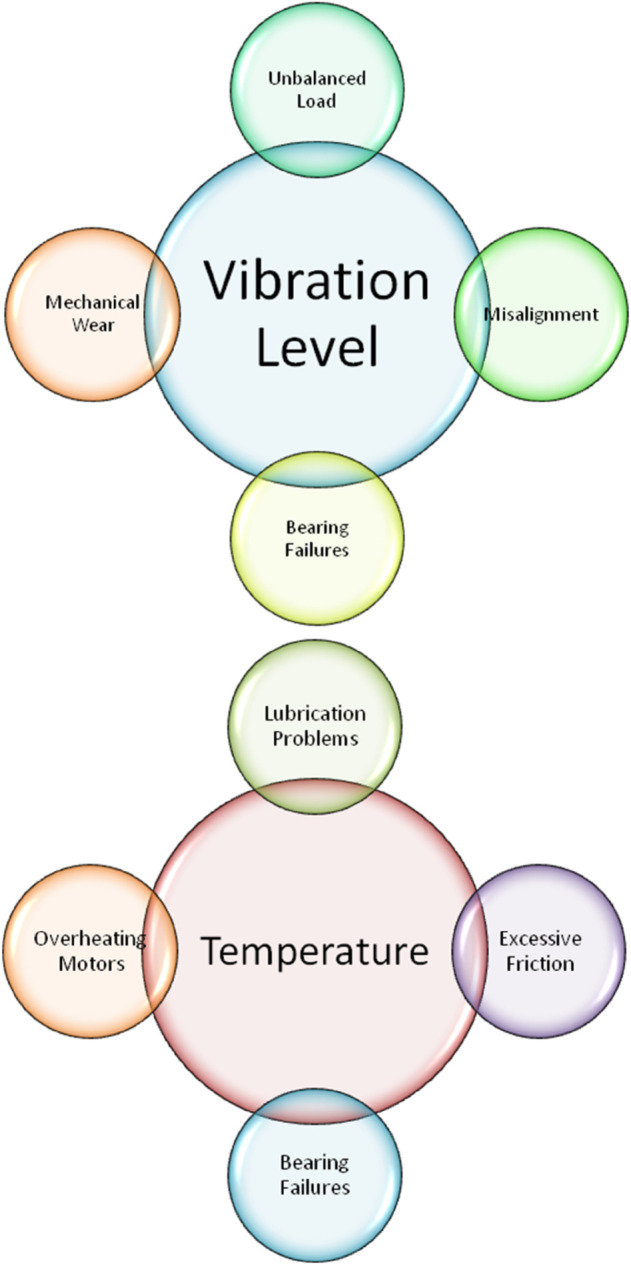
Depiction of various features of conveyor belt-I.

**Fig 7:**
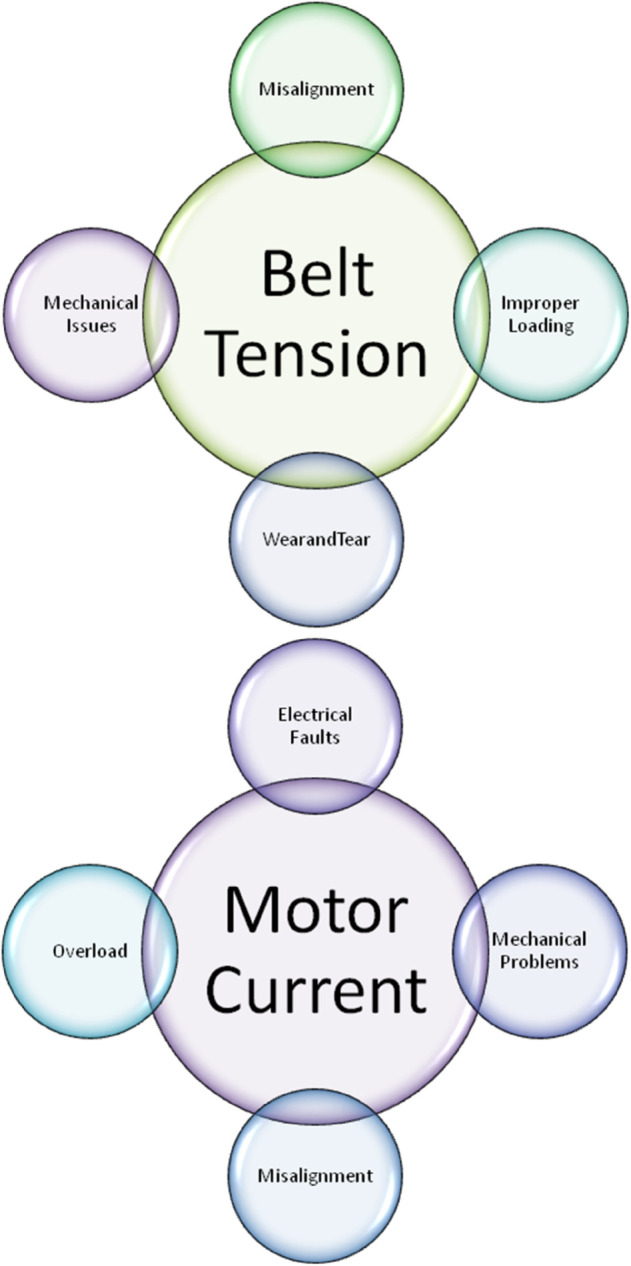
Depiction of various features of conveyor belt-II.

**Fig 8:**
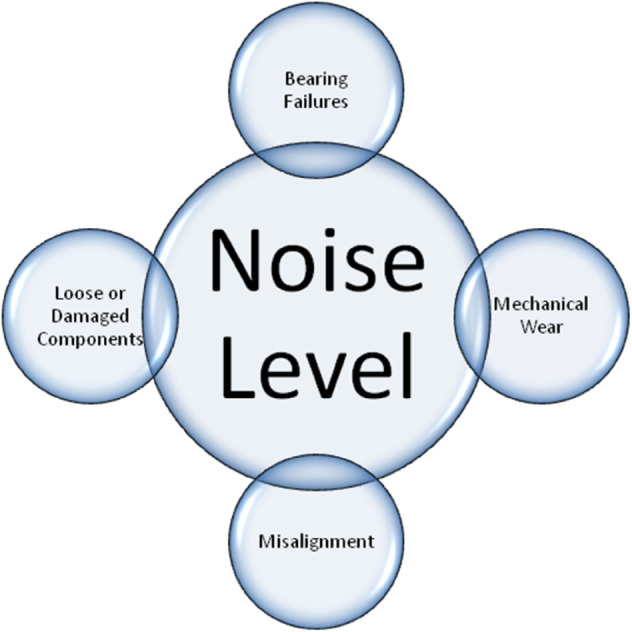
Depiction of various features of conveyor belt-III.

The features of conveyor belt have been portrayed in Figs 6, 7, and 8.

### 5.1 The algorithm for diagnosing faults in the model

This section introduces an algorithm for diagnosing unconventional faults using hypersoft fuzzy rough sets.

≪≪≪≪≪≪≪≪≪≪≪≪≪ProposedAlgorithm≫≫≫≫≫≫≫≫≫≫≫≫≫

**1. Input**

1.1. After careful analysis of existing literature and survey results, shortlist possible states of the conveyor belt system as a set of alternatives Ł.

1.2. With mutual consensus of experts, shortlist the evaluating parameters ${\hat{\alpha }}_{i}$ as a collection of parameters $\ddot{Y}$.

1.3. Enclose the sub-parametric values with respect to parameters ${\hat{\alpha }}_{i}$ in disjoint sets ${\tilde{\overset{˘}{𝔻}}}_{i}$ and find $\tilde{\overset{˘}{𝔻}}=\prod_{i}{\tilde{\overset{˘}{𝔻}}}_{i}$.

**2. Construction phase:**

2.1. Construct hypersoft fuzzy information system $\hat{T}=(\text{Ł},¥)$ for the assessment of faults.

2.2. Construct and tabulate fuzzy HSS $\left(\tilde{\Lambda },\overset{˘}{𝔻}\right)$ and its relevant pseudo fuzzy HSS $\left({\tilde{\Lambda }}^{-1},\overset{˘}{𝔻}\right)$.

**3. Computation phase:**

3.1. Compute the optimal object ${𝕁}^{+}$ and ${𝕁}^{-}$ for all the diagnosis using Eq (8) and Eq (9) respectively.

3.2. Determine the upper approximation and lower approximation of ${𝕁}^{+}$ and ${𝕁}^{-}$ concerning $\hat{T}=(\text{Ł},¥).$

3.3. Compute the score values $\xi_{\hat{ı}}\left({𝕁}^{+}\right)$ and $\xi_{\hat{ı}}\left({𝕁}^{-}\right)$ for ${𝕁}^{+}$ and ${𝕁}^{-}$ respectively using Definition 10 and Eq (10).

3.4. Compute each fault’s hypersoft close degree $\xi_{\hat{ı}}$ using Definition 11.

**4. Output phase:**

4.1. Analyze the results determined in step 3.4 and rank the alternatives.

≪≪≪≪≪≪≪≪≪≪≪≪≪≪≪≪≪≪≪≫≫≫≫≫≫≫≫≫≫≫≫≫≫≫≫

**Fig 9:**
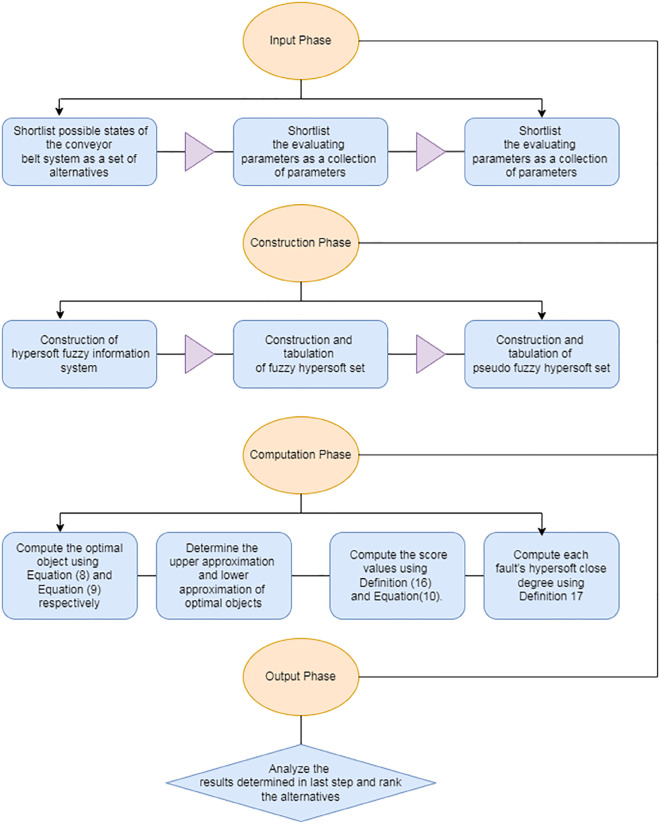
Phases of proposed algorithm.

Different phases of the proposed algorithm are stated in Fig 9. It depicts four stages: input, construction, computation, and output. The input stage is designed to provide basic requirements. The essential sets represent the basic requirements in our case. The construction stage is designed to build the basic structures necessary for the further progression of the algorithm. The computation stage includes all the calculations made during the process, while the output consists of the final results and the ranking of the alternatives.

### 5.2 Case study

The management of Ghan Group of Industries intends to find out the current working condition of conveyor belt system in its industries, its possible defects and possible solutions. A central committee comprising professionals with pertinent experience is established; some members are recruited from outside the organization, while others work for it. After thorough survey reports and existing literature review, eight possible states of the conveyor belt systems has been shortlisted as alternatives and enclosed in the set $\text{Ł}=\left\{{\overset{˘}{w}}_{1},{\overset{˘}{w}}_{2},{\overset{˘}{w}}_{3},...,{\overset{˘}{w}}_{8}\right\}$. With mutual consensus of all the members of the committee, five evaluation attributes ${\hat{\alpha }}_{1}$= vibration level, ${\hat{\alpha }}_{2}$= temperature, ${\hat{\alpha }}_{3}$= belt tension, ${\hat{\alpha }}_{4}$= motor current, and ${\hat{\alpha }}_{5}$= noise level, have been finalized for this evaluation. These parameters are enclosed in the set $\ddot{Y}=\{{\hat{\alpha }}_{1},{\hat{\alpha }}_{2},{\hat{\alpha }}_{3},{\hat{\alpha }}_{4},{\hat{\alpha }}_{5}\}$. The corresponding sub-parametric values for every attribute ${\hat{\alpha }}_{i},\;i=1,2,3,...,5$ are respectively the disjoints sets: ${\tilde{\overset{˘}{𝔻}}}_{1}=\{{\hat{\alpha }}_{11},{\hat{\alpha }}_{12},{\hat{\alpha }}_{13},{\hat{\alpha }}_{14}\}$, ${\tilde{\overset{˘}{𝔻}}}_{2}=\{{\hat{\alpha }}_{21},{\hat{\alpha }}_{22},{\hat{\alpha }}_{23},{\hat{\alpha }}_{24}\}$, ${\tilde{\overset{˘}{𝔻}}}_{3}=\{{\hat{\alpha }}_{31},{\hat{\alpha }}_{32},{\hat{\alpha }}_{33},{\hat{\alpha }}_{34}\}$, ${\tilde{\overset{˘}{𝔻}}}_{4}=\{{\hat{\alpha }}_{41},{\hat{\alpha }}_{42},{\hat{\alpha }}_{43},{\hat{\alpha }}_{44}\}$, and ${\tilde{\overset{˘}{𝔻}}}_{5}=\{{\hat{\alpha }}_{51},{\hat{\alpha }}_{52},{\hat{\alpha }}_{53},{\hat{\alpha }}_{54}\}$ respectively. Where ${\hat{\alpha }}_{11}=$ misalignment, ${\hat{\alpha }}_{12}$= unbalanced load, ${\hat{\alpha }}_{13}$= mechanical wear, ${\hat{\alpha }}_{14}$= bearing failures, ${\hat{\alpha }}_{21}$= overheating motors, ${\hat{\alpha }}_{22}$= lubrication problems, ${\hat{\alpha }}_{23}$= excessive friction, ${\hat{\alpha }}_{24}$= bearing failures, ${\hat{\alpha }}_{31}$= misalignment, ${\hat{\alpha }}_{32}$= improper loading, ${\hat{\alpha }}_{33}$= wear and tear, ${\hat{\alpha }}_{34}$= mechanical issues, ${\hat{\alpha }}_{41}$= overload, ${\hat{\alpha }}_{42}$= electrical faults, ${\hat{\alpha }}_{43}$= mechanical problems, ${\hat{\alpha }}_{44}$= misalignment, ${\hat{\alpha }}_{51}$= mechanical wear, ${\hat{\alpha }}_{52}$= misalignment, ${\hat{\alpha }}_{53}$= bearing failures, ${\hat{\alpha }}_{54}$= loose or damaged Components. To maintain the distinction of sub-parametric values, the experts filter them on preferences basis and have select whole ${\tilde{\overset{˘}{𝔻}}}_{1}$, ${\hat{\alpha }}_{21},{\hat{\alpha }}_{22}$ and ${\hat{\alpha }}_{23}$ in ${\tilde{\overset{˘}{𝔻}}}_{2}$, ${\hat{\alpha }}_{32}$, and ${\hat{\alpha }}_{33}$ in ${\tilde{\overset{˘}{𝔻}}}_{3}$, ${\hat{\alpha }}_{42}$ in ${\tilde{\overset{˘}{𝔻}}}_{4}$ and ${\hat{\alpha }}_{54}$ in ${\tilde{\overset{˘}{𝔻}}}_{5}$. After computing $\tilde{\overset{˘}{𝔻}}={\tilde{\overset{˘}{𝔻}}}_{1}\times {\tilde{\overset{˘}{𝔻}}}_{2}\times ...\times {\tilde{\overset{˘}{𝔻}}}_{5}=\{\hbar_{1},\hbar_{2},\hbar_{3},...,\hbar_{24}\}$, its subset $\hat{𝕋}=\{\hbar_{1},\hbar_{5},\hbar_{9},\hbar_{11},\hbar_{15},\hbar_{19},\hbar_{24}\}$ has been finalized for further evaluation process of diagnostic fault. Accordingly, each fault diagnosis is quantitatively described by the pseudo fuzzy binary mapping $\tilde{\Lambda }\in \Omega \left(\text{Ł}\times \overset{˘}{𝔻}\right)$ in relation to the seven multi-argument features (i.e. the fuzzy membership degree). Tables 6 and 7 present the quantitative descriptions of each fault diagnosis in relation to these attributes in fuzzy hypersoft and pseudo fuzzy hypersoft environments. Using the Formulas 8 and 9 we may derive the object plans ${𝕁}^{+}$ and ${𝕁}^{-}$, respectively, from Table 7 as follows:


$$J^{+}=\frac{0.71}{\hbar_{1}}+\frac{0.91}{\hbar_{5}}+\frac{0.91}{\hbar_{9}}+\frac{0.71}{\hbar_{11}}+\frac{0.73}{\hbar_{15}}+\frac{0.85}{\hbar_{19}}+\frac{0.71}{\hbar_{24}}.$$

$$J^{-}=\frac{0.12}{\hbar_{1}}+\frac{0.22}{\hbar_{5}}+\frac{0.26}{\hbar_{9}}+\frac{0.11}{\hbar_{11}}+\frac{0.18}{\hbar_{15}}+\frac{0.29}{\hbar_{19}}+\frac{0.18}{\hbar_{24}}.$$

**Table 6:** Tabulation of fuzzy HSS.

$\left(\tilde{\Lambda },\overset{˘}{𝔻}\right)$	${\overset{˘}{w}}_{1}$	${\overset{˘}{w}}_{2}$	${\overset{˘}{w}}_{3}$	${\overset{˘}{w}}_{4}$	${\overset{˘}{w}}_{5}$	${\overset{˘}{w}}_{6}$	${\overset{˘}{w}}_{7}$	${\overset{˘}{w}}_{8}$
$\hbar_{1}$	0.31	0.12	0.71	0.31	0.24	0.65	0.52	0.42
$\hbar_{3}$	0.43	0.24	0.35	0.43	0.72	0.91	0.22	0.22
$\hbar_{5}$	0.26	0.81	0.46	0.76	0.91	0.35	0.33	0.55
$\hbar_{6}$	0.53	0.36	0.67	0.51	0.71	0.37	0.11	0.35
$\hbar_{9}$	0.18	0.73	0.64	0.39	0.53	0.45	0.72	0.65
$\hbar_{11}$	0.42	0.76	0.53	0.83	0.78	0.73	0.29	0.85
$\hbar_{14}$	0.56	0.45	0.58	0.18	0.71	0.42	0.61	0.51

**Table 7:** Tabulation of pseudo fuzzy HSS.

$\left({\tilde{\Lambda }}^{-1},\overset{˘}{𝔻}\right)$	$\hbar_{1}$	$\hbar_{5}$	$\hbar_{9}$	$\hbar_{11}$	$\hbar_{15}$	$\hbar_{19}$	$\hbar_{24}$
${\overset{˘}{w}}_{1}$	0.31	0.43	0.26	0.53	0.18	0.42	0.56
${\overset{˘}{w}}_{2}$	0.12	0.24	0.81	0.36	0.73	0.76	0.45
${\overset{˘}{w}}_{3}$	0.71	0.35	0.46	0.67	0.64	0.53	0.58
${\overset{˘}{w}}_{4}$	0.31	0.43	0.76	0.51	0.39	0.83	0.18
${\overset{˘}{w}}_{5}$	0.24	0.72	0.91	0.71	0.53	0.78	0.71
${\overset{˘}{w}}_{6}$	0.65	0.91	0.35	0.37	0.45	0.73	0.42
${\overset{˘}{w}}_{7}$	0.52	0.22	0.33	0.11	0.72	0.29	0.61
${\overset{˘}{w}}_{8}$	0.42	0.22	0.55	0.35	0.65	0.85	0.51

Next, we may use Formula 8 to determine the upper and lower approximations of object plan ${𝕁}^{+}$, or the hypersoft close degree, for each fault diagnosis.

The same procedure can be repeated for ${𝕁}^{-}.$ The upper and lower approximations of ${𝕁}^{+}$ and ${𝕁}^{-}$ are tabulated in Table 8 and Table 9 respectively. Based on the values of Tables 8 and 9, fault’s hypersoft close degrees $\xi_{\hat{ı}}$ are determined that are tabulated in Table 10.

**Table 8:** Tabulation of upper and lower approximations of object plan ${𝕁}^{+}$.

	${\overset{˘}{w}}_{1}$	${\overset{˘}{w}}_{2}$	${\overset{˘}{w}}_{3}$	${\overset{˘}{w}}_{4}$	${\overset{˘}{w}}_{5}$	${\overset{˘}{w}}_{6}$	${\overset{˘}{w}}_{7}$	${\overset{˘}{w}}_{8}$
$\overset{\leftarrow }{\Omega }\left({𝕁}^{+}\right)$	0.71	0.71	0.71	0.71	0.71	0.71	0.71	0.71
$\vec{\Omega }\left({𝕁}^{+}\right)$	0.56	0.81	0.71	0.83	0.91	0.91	0.72	0.85
$\xi_{\hat{ı}}\left({𝕁}^{+}\right)$	1.27	1.52	1.42	1.54	1.62	1.62	1.43	1.56

**Table 9:** Tabulation of upper and lower approximations of object plan ${𝕁}^{-}$.

	${\overset{˘}{w}}_{1}$	${\overset{˘}{w}}_{2}$	${\overset{˘}{w}}_{3}$	${\overset{˘}{w}}_{4}$	${\overset{˘}{w}}_{5}$	${\overset{˘}{w}}_{6}$	${\overset{˘}{w}}_{7}$	${\overset{˘}{w}}_{8}$
$\overset{\leftarrow }{\Omega }\left({𝕁}^{-}\right)$	0.44	0.26	0.29	0.29	0.26	0.22	0.28	0.29
$\vec{\Omega }\left({𝕁}^{-}\right)$	0.29	0.29	0.29	0.29	0.29	0.29	0.29	0.29
$\xi_{\hat{ı}}\left({𝕁}^{-}\right)$	0.73	0.55	0.58	0.58	0.55	0.51	0.57	0.58

**Table 10:** Tabulation of fault’s hypersoft close degree $\xi_{\hat{ı}}$.

	${\overset{˘}{w}}_{1}$	${\overset{˘}{w}}_{2}$	${\overset{˘}{w}}_{3}$	${\overset{˘}{w}}_{4}$	${\overset{˘}{w}}_{5}$	${\overset{˘}{w}}_{6}$	${\overset{˘}{w}}_{7}$	${\overset{˘}{w}}_{8}$
$\xi_{\hat{ı}}\left({𝕁}^{+}\right)$	1.27	1.52	1.42	1.54	1.62	1.62	1.43	1.56
$\xi_{\hat{ı}}\left({𝕁}^{-}\right)$	0.73	0.55	0.58	0.58	0.55	0.51	0.57	0.58
$\xi_{\hat{ı}}$	0.54	0.97	0.84	0.94	1.07	1.11	0.86	0.98

**Fig 10:**
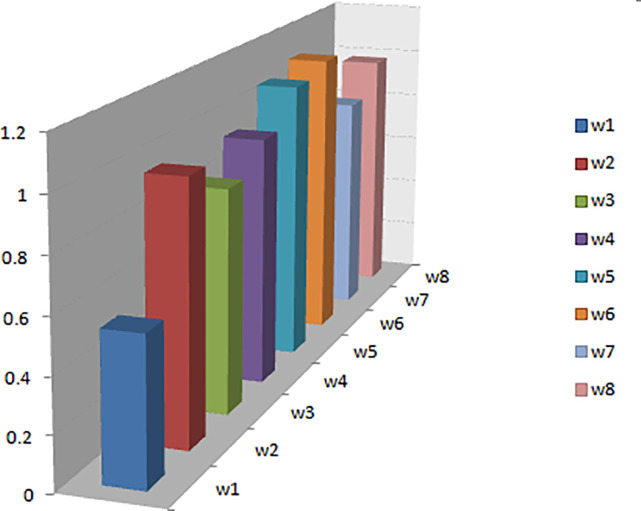
Ranking of alternatives based on $\xi_{\hat{ı}}$.

According to fault’s hypersoft close degrees $\xi_{\hat{ı}}$, the alternatives are ranked as ${\overset{˘}{w}}_{6}>{\overset{˘}{w}}_{5}>{\overset{˘}{w}}_{8}>{\overset{˘}{w}}_{2}>{\overset{˘}{w}}_{4}>{\overset{˘}{w}}_{7}>{\overset{˘}{w}}_{3}>{\overset{˘}{w}}_{1}$. It can easily be observed that the alternative ${\overset{˘}{w}}_{6}$ received the highest score, therefore, it is opted for final selection. The ranking of all the alternatives is presented in Fig 10. Regarding the sensitivity analysis of the results obtained, in the context of our current work, it is not directly applicable due to the nature and structure of our proposed methodology. The parameters and assumptions used in our approach are either fixed or determined through established criteria, leaving limited room for variability that would significantly benefit from sensitivity analysis.

### 5.3 Discussion and comparison

In this paper, we investigate the use of pseudo fuzzy hypersoft rough approximations in industrial defect diagnostics, considering the dynamic and intricate nature of contemporary industrial systems. These systems often consist of many interconnected parts, which behave erratically and imprecisely due to factors such as operational uncertainty. While traditional fault detection techniques perform well in simpler situations, they struggle to comprehend and assess the complex relationships and ambiguous data present in these environments. By integrating the advantages of fuzzy logic, hypersoft sets, and rough approximations, pseudo fuzzy hypersoft rough approximations offer a robust and flexible framework for handling uncertainty, imprecision, and interdependence. This methodology enhances diagnostic accuracy and reliability in industrial contexts by promoting a more sophisticated understanding of defect patterns. Since the defect diagnosis has not yet been discussed in the literature using parameterization tools, multi-argument domain settings, or rough approximations settings, the ranking-based comparison of the proposed framework is in fact impracticable. However, by considering a few important factors, its adaptability can be evaluated. This comparison is shown in Table 11, which indicates that while the currently available references are somewhat inadequate, the proposed framework is adequate for all such assessing factors.

**Table 11:** Comparison analysis.

Scholars	Frameworks	Multi-argument domain settings	Rough approximations	Modeling uncertainties using fuzzy membership grades	Parameterization tool	Fault diagnosis
Cayrac *et al*. [13]	Possibility FS	Deficient	Deficient	Sufficient	Deficient	Sufficient
Feng *et al*. [38]	SRS	Deficient	Sufficient	Deficient	Sufficient	Deficient
Ali [39]	RSS	Deficient	Sufficient	Deficient	Sufficient	Deficient
Shabir *et al*. [40]	SRS	Deficient	Sufficient	Deficient	Sufficient	Deficient
Sun and Ma [43]	SFRS	Deficient	Sufficient	Sufficient	Sufficient	Deficient
Meng *et al*. [44]	SFRS	Deficient	Sufficient	Sufficient	Sufficient	Deficient
Hu *et al*. [45]	SFRS	Deficient	Sufficient	Sufficient	Sufficient	Deficient
Zhang [48]	RSS	Deficient	Sufficient	Deficient	Sufficient	Deficient
Proposed Framework	HFRS	Sufficient	Sufficient	Sufficient	Sufficient	Sufficient

This study offers a number of noteworthy benefits (given below), proving its worth in developing problem diagnosis techniques for intricate industrial systems.:

1. The approach successfully manages ambiguity, vagueness, and granularity by combining fuzzy rough set theory with hypersoft sets, which makes it ideal for intricate defect identification situations.
2. The suggested algorithm provides an organized method for prudent decision-making in business environments by utilizing the modified Bingzhen and Weimin decision model.
3. The preciseness of diagnostic results is improved by introducing hypersoft fuzzy rough lower and upper approximation operators, which allow for sophisticated appraisals of fuzzy subsets within parameter sets.
4. The flexibility of the proposed framework in handling complicated systems is demonstrated by its capacity to analyze a large number of 20 sub-parameters and important 5 evaluating parameters.
5. By evaluating eight possible fault states according to fuzzy hypersoft close degrees, a hypothetical case study for conveyor belt systems demonstrates the potential of proposed algorithm in practical industrial applications and proves its dependability. The fuzzy hypersoft close degrees enable decision-makers prioritize remedies by offering an explicit and organized rating of fault states.

## 6 Conclusions

This study presents a novel framework for fault diagnosis in industrial systems based on the integration of hypersoft sets and fuzzy rough set theory. The proposed hypersoft fuzzy rough set model effectively addresses uncertainty, vagueness, and the hierarchical nature of multi-attribute parameters. We developed and applied new lower and upper approximation operators using pseudo fuzzy binary relations, resulting in improved classification and decision-making accuracy. The model has been evaluated through a real-world-inspired case study and a simulation-based comparison with existing techniques, both of which confirmed its robustness and effectiveness. Furthermore, a sensitivity analysis validated the model’s stability under parameter fluctuations. This work contributes significantly to the field of decision-making under uncertainty by extending hypersoft set theory in a novel and practical direction. The suggested strategy performed better than conventional techniques by successfully managing uncertainty and capturing the subtleties of imprecise data. More accurate fault identification was made possible by the rich representation of multi-attribute data made possible by the hierarchical structure of hypersoft sets. Rough sets offered a way to approximate ambiguous data and discriminate between states that were unquestionably faulty and those that may be problematic. In industrial systems, where it is frequently impractical to make sharp distinctions, this capacity is essential. The suggested approach showed adaptability, working with a range of industrial systems such as motors, conveyor belts, and intricate manufacturing configurations. It is a useful tool for industrial fault diagnostics because to its context-adaptability. The proposed model holds strong potential for application in a variety of domains beyond fault diagnosis in conveyor systems. It can be employed in predictive maintenance for manufacturing and automotive systems, early fault detection in power plants and energy systems, and anomaly identification in sensor-driven environments such as IoT-based smart factories. Moreover, its adaptability to uncertain, multi-layered decision environments makes it suitable for risk assessment in construction, aerospace fault classification, and quality control in automated production lines. The model’s ability to integrate interrelated attributes and handle vagueness positions it as a versatile tool in modern industrial analytics.

Despite the promising results, the proposed model has some limitations. The current study assumes static parameter weights, which may not capture real-time shifts in industrial processes. Additionally, while the model handles uncertainty and vagueness effectively, its performance may vary with different types of fuzzy membership functions or more complex interdependencies among parameters. Future research can focus on dynamic parameter modeling, integration with machine learning for adaptive decision-making, and real-time data stream handling. To improve diagnostic accuracy, future studies can concentrate on improving the models by adding more layers of parameters and investigating various kinds of fuzzy membership functions. Rough hypersoft sets and machine learning algorithms can be combined to generate hybrid models that include the best features of both techniques. Systems for defect diagnosis that are even more precise and effective may result from such integration. One possible avenue is the development of real-time diagnostic systems based on rough hypersoft sets. Putting these technologies into operation in real-world industrial settings might yield insightful criticism and chances for more improvement. Moreover, testing the model in other high-risk industrial environments such as aerospace, oil and gas, or smart grid systems could further validate and enhance its practical utility.
